# Aptamer-Based Nongenetic Reprogramming of CARs Enables Flexible Modulation of T Cell-Mediated Tumor Immunotherapy

**DOI:** 10.1021/acscentsci.3c01511

**Published:** 2024-03-21

**Authors:** Qiang Zhang, Limei Wu, Yue Zhang, Dan Wang, Yingyu Sima, Zhimin Wang, Zhiwei Yin, Hui Wu, Yuting Zhuo, Yutong Zhang, Linlin Wang, Yong Chen, Yanlan Liu, Liping Qiu, Weihong Tan

**Affiliations:** † Molecular Science and Biomedicine Laboratory (MBL), State Key Laboratory of Chemo/Biosensing and Chemometrics, College of Chemistry and Chemical Engineering, College of Biology, Aptamer Engineering Center of Hunan Province, Hunan University, Changsha, Hunan 410082, P. R. China; ‡ The Key Laboratory of Zhejiang Province for Aptamers and Theranostics, Zhejiang Cancer Hospital, Hangzhou Institute of Medicine (HIM), Chinese Academy of Sciences, Hangzhou, Zhejiang 310022, P. R. China; § Institute of Molecular Medicine (IMM), Renji Hospital, Shanghai Jiao Tong University School of Medicine, and College of Chemistry and Chemical Engineering, Shanghai Jiao Tong University, Shanghai 200240, P. R. China; ∥ NHC Key Laboratory of Birth Defect for Research and Prevention, Hunan Provincial Maternal and Child Health Care Hospital, Changsha 410000, P. R. China

## Abstract

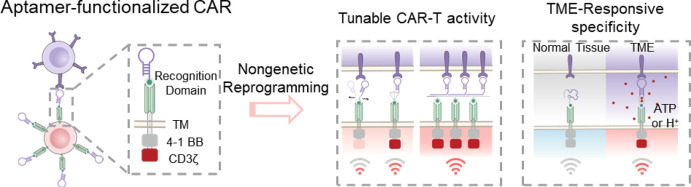

Innovating the design of chimeric antigen receptors (CARs) beyond conventional structures would be necessary to address the challenges of efficacy, safety, and applicability in T cell-based cancer therapy, whereas excessive genetic modification might complicate CAR design and manufacturing, and increase gene editing risks. In this work, we used aptamers as the antigen-recognition unit to develop a nongenetic CAR engineering strategy for programming the antitumor activity and specificity of CAR T cells. Our results demonstrated that aptamer-functionalized CAR (Apt-CAR) T cells could be directly activated by recognizing target antigens on cancer cells, and then impart a cytotoxic effect for cancer elimination in vitro and in vivo. The designable antigen recognition capability of Apt-CAR T cells allows for easy modulation of their efficacy and specificity. Additionally, multiple features, e.g., tunable antigen-binding avidity and the tumor microenvironment responsiveness, could be readily integrated into Apt-CAR design without T cell re-engineering, offering a new paradigm for developing adaptable immunotherapeutics.

## Abstract 2

We used aptamers as the antigen-targeting unit and developed a nongenetic CAR reprograming platform for customized modulation of T cell-based cancer immunotherapy. Based on the excellent programmability of DNA nanotechnology and the designable recognition capability of aptamers, the cancer targeting avidity and specificity of aptamer-functionalized CAR (Apt-CAR) T cells can be readily modulated, offering a new paradigm for the development of modulable immunotherapeutics.

## Introduction

Chimeric antigen receptors (CARs) are synthetic transmembrane proteins that comprise an extracellular domain for antigen recognition and intracellular signaling domains derived from T-cell receptors and costimulatory molecules.^1^ With impressive outcomes in treating B cell malignancies, CAR T cells have sparked a revolution in cancer immunotherapy.^2^ Nevertheless, their applicability to other types of cancer is generally challenged by safety concerns, suboptimal efficacy, and complex manufacturing processes.^3,4^ Particularly, due to the paucity of tumor-specific antigens and the immunosuppressive tumor microenvironment, additional obstacles are posed in battle against solid tumors.^5^ Innovating the design of CARs beyond that of conventional structures is necessary to address these challenges.

Numerous CAR engineering strategies have been developed, such as CARs for bivalent antigen recognition,^6^ small molecule-triggered CARs,^7^ and light-responsive CARs,^8^ to improve the specificity and efficacy of T-cell therapy.^9^ Despite their promising potential, these advanced CARs require excessive genetic modification,^10^ which not only increases the complexity of CAR design and production, but also intensifies the risks of gene editing.^4^ Besides, owing to the significant overlap in antigen profiles between solid tumors and normal tissues,^11^ it remains challenging to select optimal antigens and their combinations to define tumors.^12^ Nevertheless, tumor development involves a sequence of physicochemical changes that lead to the restructuring of the tumor microenvironment (TME), which has unique characteristics distinct from normal tissues.^13^ Incorporating the hallmarks of the TME into CAR design is expected to mitigate the on-target off-tumor toxicity.^14^ Indeed, approaches that facilitate the incorporation of multiple control elements into CAR programming and alleviate the requirement for extensive genetic optimization are highly desirable.

To exert control over the CAR performance without involving genetic modification, tunable antigen recognition units would be potentially useful.^15^ Aptamers, single-stranded oligonucleotides capable of specifically binding with target molecules via folding into given secondary/tertiary configuration, are particularly suited for this purpose.^16^ The cell-SELEX (Systematic Evolution of Ligands by Exponential Enrichment) technology allows screening a panel of aptamers against molecular signatures of target cells, thus enriching the toolbox for antigen recognition.^17^ Furthermore, the intrinsic properties of nucleic acids make aptamers easily integrable with DNA nanotechnology, providing diverse opportunities for manipulating antigen recognition events.^18^ While aptamers have been demonstrated to enhance cancer-specific binding of T cells,^19−21^ the potential of aptamer-based CAR constructs has not been extensively explored.

**Figure 1:**
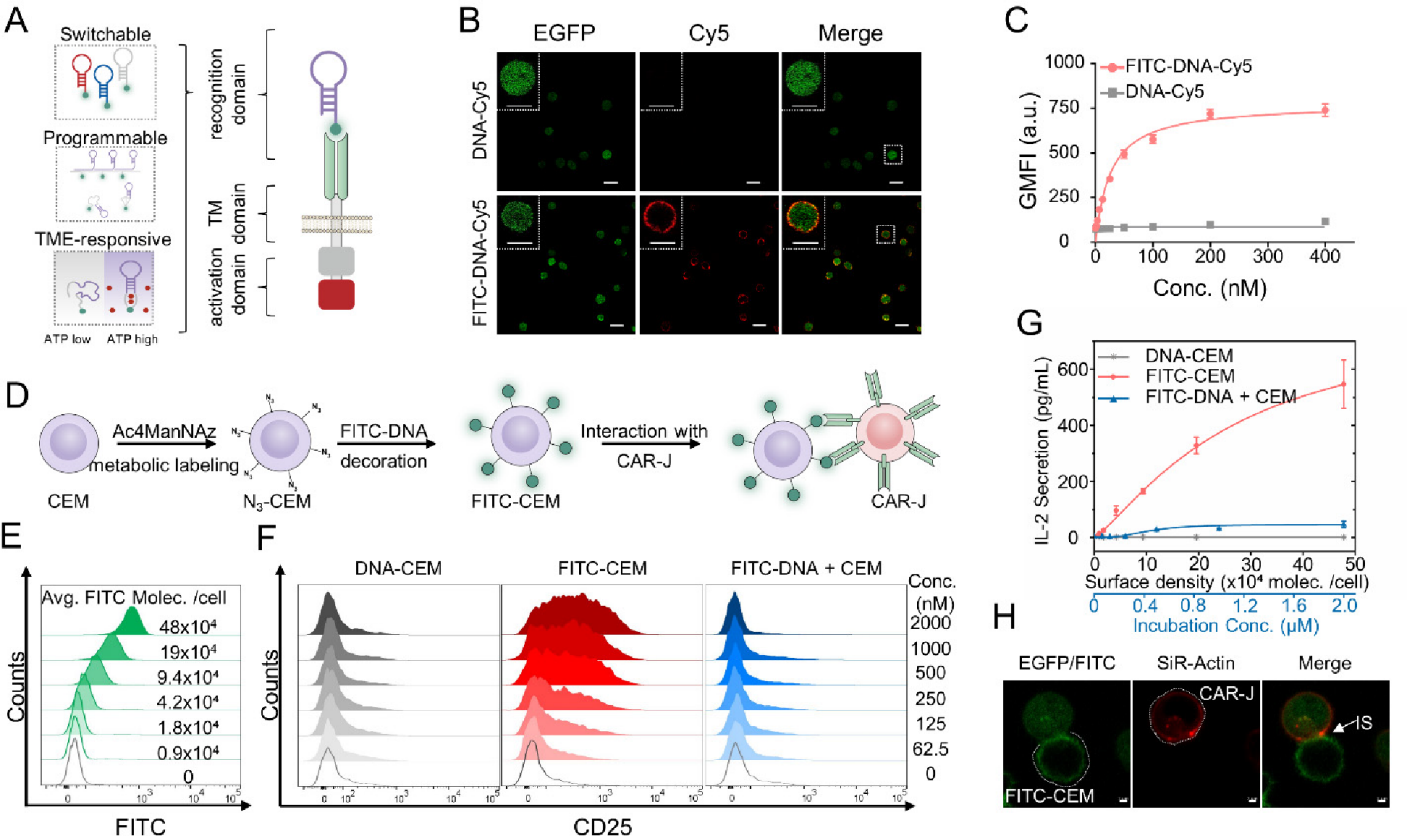
Construction and characterization of anti-FITC CAR-expressing Jurkat T cells (CAR-J). (A) Structures of aptamer-functionalized CAR. The recognition domain was incorporated with switchable, programmable, and TME-responsive (via adenosine triphosphate [ATP] expression) aptamers. (B) CLSM images of CAR-J treated with 50 nM DNA-Cy5 or FITC-DNA-Cy5 in binding buffer at 4 °C for 30 min. Scale bars in the whole images and the enlarged view represent 20 and 10 μm, respectively. (C) Plotting Cy5 fluorescence intensity of DNA-Cy5 or FITC-DNA-Cy5-treated CAR-J versus DNA concentration, as assayed with flow cytometry. (D) Schematic illustration of constructing FITC-DNA-conjugated CEM (FITC-CEM) cells and their interaction with CAR-J. (E) Flow cytometry analysis of Ac4ManNAz-pretreated CEM cells after incubation with FITC-DNA-DBCO with different concentrations in binding buffer at 37 °C for 1 h. The cell surface density of FITC moiety was calculated according to the FITC fluorescence of the cell lysates. (F) CD25, an immune regulatory molecule, expressing CAR-J after coculture with CEM cells conjugated with DNA of different density (DNA-CEM), CEM cells conjugated with FITC-DNA of different density (FITC-CEM), or CEM cells plus free FITC-DNA of different concentrations (FITC-DNA + CEM) at 37 °C for 24 h, as assayed with flow cytometry. (G) IL-2 secretion of CAR-J in corresponding samples of F, as assayed with BD CBA cytokine kit. (H) CLSM images of CAR-J (stained with SiR-Actin Kit) interacting with FITC-CEM. White arrowhead points to the IS, the nanoscale gap between T cells and antigen presenting cells. Scale bars represent 2 μm. All flow cytometric diagrams are representative data from three independent experiments. All statistical data are presented as the mean value ± S.D., *n* = 3.

In this work, we developed an aptamer-based nongenetic CAR engineering strategy for programming the antitumor activity of T cells (Figure 1A). Briefly, we designed a switchable CAR construct that consisted of a single-chain variable fragment (scFv) incorporated with an aptamer for antigen recognition. Via aptamer-mediated specific binding to target antigens on cancer cells, CAR T cells could be directly activated and subsequently exert cytotoxic effects against the cancer cells. By combing designable recognition capability of aptamers with programmable DNA nanotechnology,^22−24^ the antigen accessibility and binding affinity of CAR T cells could be readily modulated. Moreover, our aptamer-functionalized CAR (Apt-CAR) enabled TME-responsive antigen recognition without the need for T cell re-engineering, paving a new way to address the safety concern of adoptive cell therapy.

## Results and Discussion

### Construction and Characterization of Aptamer-Functionalized CARs

To construct Apt-CAR T cells, an anti-FITC (fluorescein isothiocyanate)-E2 scFv fragment was fused with a second-generation CAR containing a CD8α hinge, a CD8α transmembrane (TM) fragment, a 4–1BB costimulatory fragment, and a CD3ζ activation fragment in the intracellular domain (Figure S1 of the Supporting Information).^25^ To indicate the expression of anti-FITC CAR, enhanced green fluorescent protein (EGFP) was noncovalently linked at the C-terminus with a Thosea asigna virus (T2A) ribosomal skip element, which could rule out any possible interference of EGFP on the CAR function. The performance of this anti-FITC CAR design was first evaluated on immortalized Jurkat T cells (Figure S2).^26^ As assayed with flow cytometry, a significant EGFP fluorescence was observed on anti-FITC CAR-transfected Jurkat T cells (termed as CAR-J, Figure S3A). The successful construction of CAR-J and the effective cleavage of the T2A linker was confirmed with Western blotting (Figure S3B). To endow CAR-J with antigen-targeting capability, one end of the aptamer was labeled with an FITC moiety, termed as Apt. Notably, the FITC moiety was exclusively employed as a binding tag for the anti-FITC CAR construct, rather than as a fluorescence reporter. The Apt functionalization was initially assessed by treating CAR-J with a FITC-DNA-Cy5, a DNA strand double-labeled with an FITC moiety and a Cyanine5 (Cy5) fluorophore. As imaged with confocal laser scanning microscopy (CLSM), a significant Cy5 fluorescence was observed on the membrane of CAR-J after treatment with FITC-DNA-Cy5 (Figures 1B and S4). The outer-membrane orientation of DNA was also verified by the scant fluorescence left after treating the CAR-J with DNase I (Figure S5). Meanwhile, a high binding affinity of FITC-DNA-Cy5 against CAR-J (the dissociation constant *K*_d_ = 26.38 nM) was achieved (Figure 1C). In contrast, CAR-J treated with FITC-deficient DNA-Cy5 or Ctrl-J (blank lentiviral vector-transfected Jurkat cells) treated with FITC-DNA-Cy5 showed negligible Cy5 fluorescence (Figure S6), indicating the FITC-based functionalization of DNA probes on CAR-J.

To evaluate the feasibility of this CAR construct for antigen recognition-directed T cell activation, we first built an artificial antigen-expressing cancer cell model. In brief, CEM cells were processed with *N*-azidoacetylmannosamine-tetraacylated (Ac4ManNAz), an azide-containing metabolic glycoprotein labeling reagent (Figure 1D), followed by covalent conjugation with DBCO-labeled FITC-DNA (FITC-DNA-DBCO) via copper-free click chemistry, termed FITC-CEM. As assayed with flow cytometry, the FITC fluorescence profile of CEM cells was gradually shifted by increasing the concentration of FITC-DNA-DBCO (Figures 1E and S7A), matching well with the gradually enhanced fluorescence on the cell surface (Figure S7B), indicating the successful construction of cancer cells containing artificial antigen of different surface densities (Figure S8). After incubation with FITC-CEM, the expression level of CD25 and CD69, two T cell activation markers,^7^ on CAR-J was significantly enhanced, showing a positive correlation with the surface density of the FITC moiety (Figures 1F and S9). In contrast, negligible upregulation of these two markers could be detected in CAR-J treated with either FITC-deficient DNA-modified CEM cells (DNA-CEM) or CEM plus soluble FITC-DNA. Similar results were obtained in the secretion of interleukin-2 (IL-2) (Figure 1G), revealing that CAR-J could be activated by specific interaction with cell surface-immobilized antigens, rather than free antigens in solution. In addition, recruitment of actin to the interface between CAR-J and FITC-CEM was observed, revealing that T cells were activated through antigen recognition-induced formation of immunological synapse (IS) (Figures 1H and S10), as consistent with previous reports.^27^

We next tested whether aptamer-mediated recognition of cancer cells could activate CAR-J. As a proof-of-concept, an aptamer specific for tumor-associated EpCAM antigen was chosen as the recognition ligand and labeled with a FITC moiety (termed Apt_EpCAM_).^28^ The specific binding of Apt_EpCAM_ against EpCAM^+^ MDA-MB-231 cells (termed 231), but not EpCAM^–^ Jurkat T cells, was first verified with flow cytometry (Figure S11). By coculturing Apt_EpCAM_-functionalized CAR-J (Apt_EpCAM_-CAR-J) with 231 cells at 37 °C for 24 h, the IL-2 secretion of T cells was significantly enhanced in an Apt concentration-dependent manner (Figure S12). Alternatively, a comparatively lower IL-2 level was detected in the control group of Lib-functionalized CAR-J (Lib-CAR-J), in which the aptamer sequence was replaced with a random one (Lib). Meanwhile, significantly lower IL-2 secretion was induced by treating CAR-J with Apt_EpCAM_ only, indicating that the aptamer functionalization process did not stimulate CAR-J. Besides, the CD25 expression of Apt_EpCAM_-CAR-J was significantly enhanced by coculture with 231 cells (Figure S13). These results verified that CAR-J could be activated through aptamer-based specific binding of cancer cells.

### Aptamer-Mediated Cancer Cell-Directed Activation of Primary CAR T Cells

**Figure 2:**
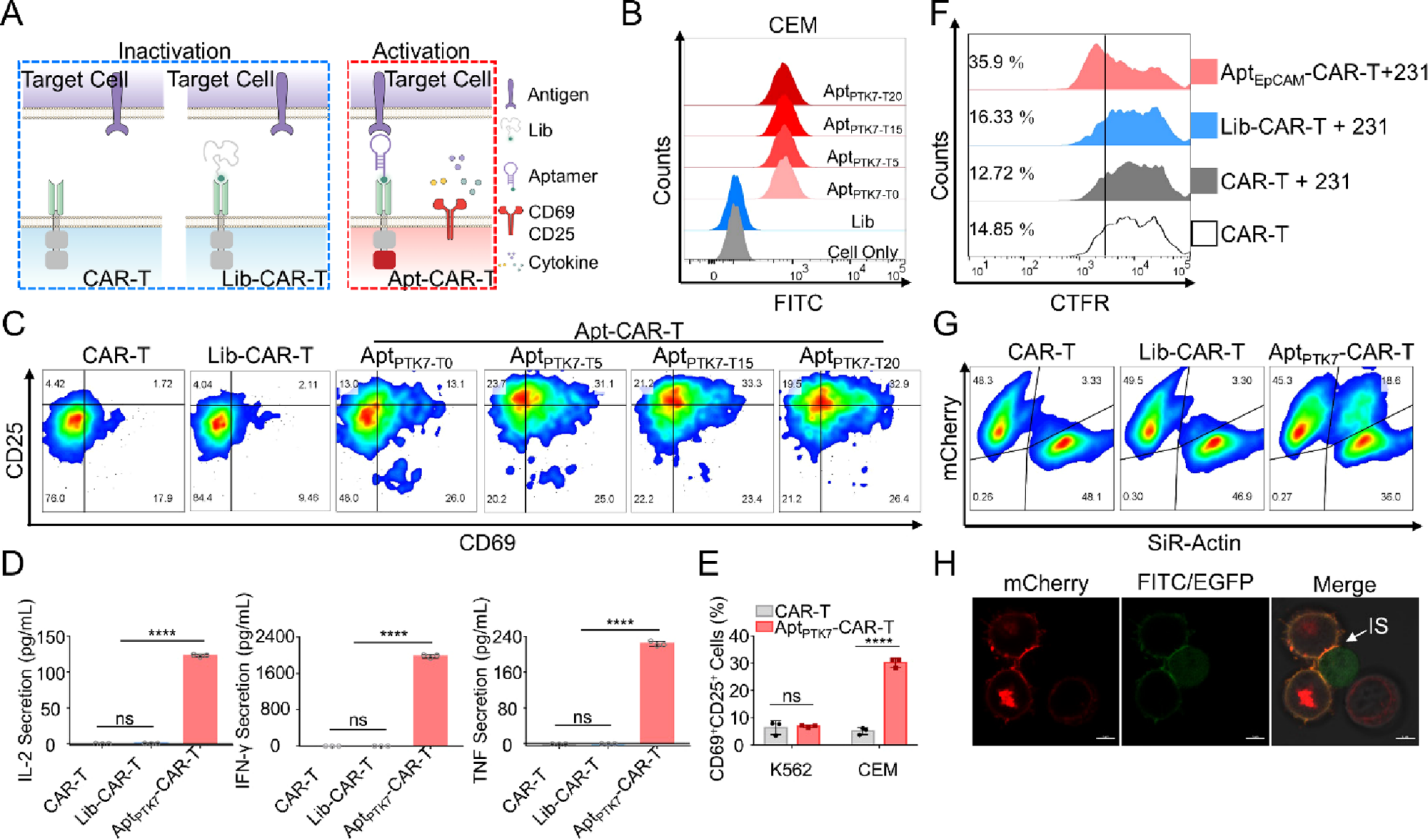
Aptamer-mediated cancer cell-directed activation of CAR T cells. (A) Schematic illustration of aptamer-mediated CAR-T activation. (B) Flow cytometry of CEM cells after incubation with 200 nM Lib, Apt_PTK7-T0_, Apt_PTK7-T5_, Apt_PTK7-T15_, or Apt_PTK7-T20_ in binding buffer at 4 °C for 30 min. (C) CD69 and CD25 expression of CAR-T and CAR-T functionalized with Lib, Apt_PTK7-T0_, Apt_PTK7-T5_, Apt_PTK7-T15_, or Apt_PTK7-T20_ after coculture with CEM at 37 °C for 24 h, as assayed with flow cytometry. (D) Secretion of IL-2, IFN-γ, and TNF of different CAR-T after incubation with CEM at 37 °C for 24 h, as assayed with BD CBA cytokine kit. (E) CD69 and CD25 expression of CAR-T and Apt_PTK7_-CAR-T after incubation with target PTK7^+^ CEM cells or nontarget PTK7^–^ K562 cells at 37 °C for 24 h, as assayed with flow cytometry. (F) Flow cytometry analysis of Apt_EpCAM_-functionalized CAR-T (Apt_EpCAM_-CAR-T), Lib-CAR-T, or CAR-T after coculture with target 231 cells for 3 days. All CAR-T cells were prestained with CellTrace Far Red (CTFR). (G) Flow cytometry analysis of PTK7 overexpressed K562 cells after incubation with CAR-T, Lib-CAR-T, or Apt_PTK7_-CAR-T. T cells were prestained with SiR-Actin kit. (H) CLSM imaging of Apt_PTK7_-CAR-T interacting with PTK7 overexpressed K562. White arrowhead points to the IS. Scale bars represent 5 μm. All flow cytometric diagrams are representative data from three independent experiments. All statistical data are presented as the mean value ± S.D., *n* = 3. *P ≤ 0.05, **P ≤ 0.01, ***P ≤ 0.001, and ****P ≤ 0.0001 by two-tailed Student’s *t* test. For all primary CAR T cell testing, three independent experiments were performed using human peripheral blood mononuclear cells (PBMCs) collected from at least two donors.

We next tested the performance of Apt-CAR on primary human T cells (Figure 2A). CD3^+^ T cells were isolated from human peripheral blood mononuclear cells (PBMCs, Figure S14) and transfected with anti-FITC CAR (the resultant T cell was termed CAR-T). As indicated by the double positive signal of EGFP and Cy5 (from FITC-DNA-Cy5), the cellular transfection efficiency of anti-FITC CAR was about 35% (Figure S15). Anti-PTK7 aptamer (Apt_PTK7_), which could specifically bind PTK7^+^ CEM cells, was chosen as the targeting unit (Figures 2B and S16).^29^ Since the linker length between the antigen-binding domain and the transmembrane domain was important for CAR function,^30^ we synthesized four anti-PTK7 aptamers with a poly-T linker of 0 nt, 5 nt, 15 nt, and 20 nt, termed as Apt_PTK7-T0_, Apt_PTK7-T5_, Apt_PTK7-T15_, and Apt_PTK7-T20_, respectively. By coculturing with CEM cells, over 13% CD69^+^CD25^+^ T cells were obtained in these four Apt_PTK7_-functionalized CAR-T groups, and the Apt_PTK7-T15_ group performed the best (33.3%, Figure 2C), whereas less than 3% of CD69^+^CD25^+^ T cells could be detected in the control groups of Lib-functionalized CAR-T (Lib-CAR-T). With its optimal performance, Apt_PTK7-T15_-functionalized CAR-T was used as the optimal desgin and simply termed as Apt_PTK7_-CAR-T in subsequent studies.

In addition to membrane marker expression, the secretion of proinflammatory cytokines, including tumor necrosis factor (TNF), IL-2, and interferon-γ (IFN-γ), were significantly enhanced by incubating Apt_PTK7_-CAR-T with target PTK7^+^ CEM, while little cytokine secretion was observed in the control group of Lib-CAR-T (Figures 2D and S17). Additionally, by coculturing with PTK7^–^ K562 cells, Apt_PTK7_-CAR-T cells exhibited a relatively lower level of cytokine secretion, as well as reduced expression of CD69 and CD25 (Figures 2E and S18). The universality of this Apt-CAR design for target cancer cell-directed T cell activation was further confirmed in different combination of aptamers and cancer cells, including anti-CD71 aptamer (Apt_CD71_) against CD71^+^ CEM cells (Figure S19),^31^ and Apt_EpCAM_ against 231 cells (Figure S20) but not EpCAM^–^ HeLa cells (Figure S21).

Being a customized living drug, the expansion of CAR-T cells in response to antigen stimulation is considered a crucial factor in achieving a complete response.^32^ The CAR-T proliferation was evaluated by CTFR dilution-based flow cytometry assay. After coculture with mitomycin C-pretreated 231 cells,^7^ the proliferation rate of Apt_EpCAM_-CAR-T (35.9%) was significantly higher than that of Lib-CAR-T (16.3%), demonstrating that aptamer-based cancer targeting could enhance the expansion efficiency of CAR-T cells (Figure 2F). To further characterize the role of aptamer-antigen binding in CAR-T stimulation, K562 cells were transfected with mCherry-PTK7 fusion protein (termed PTK7^+^ K562, Figure S22). Apt_PTK7_-induced specific contact between CAR-T and PTK7^+^ K562 was first confirmed with flow cytometry (Figure 2G). Meanwhile, the fluorescence of mCherry and FITC was colocalized at the interface between these two cells (Figure 2H), revealing that aptamer-mediated antigen binding could promote the aggregation of ligated CAR constructs to form IS,^33,34^ thus consequently leading to effective T cell activation.

### Aptamer-Mediated Specific Elimination of Cancer Cells by CAR-T

**Figure 3:**
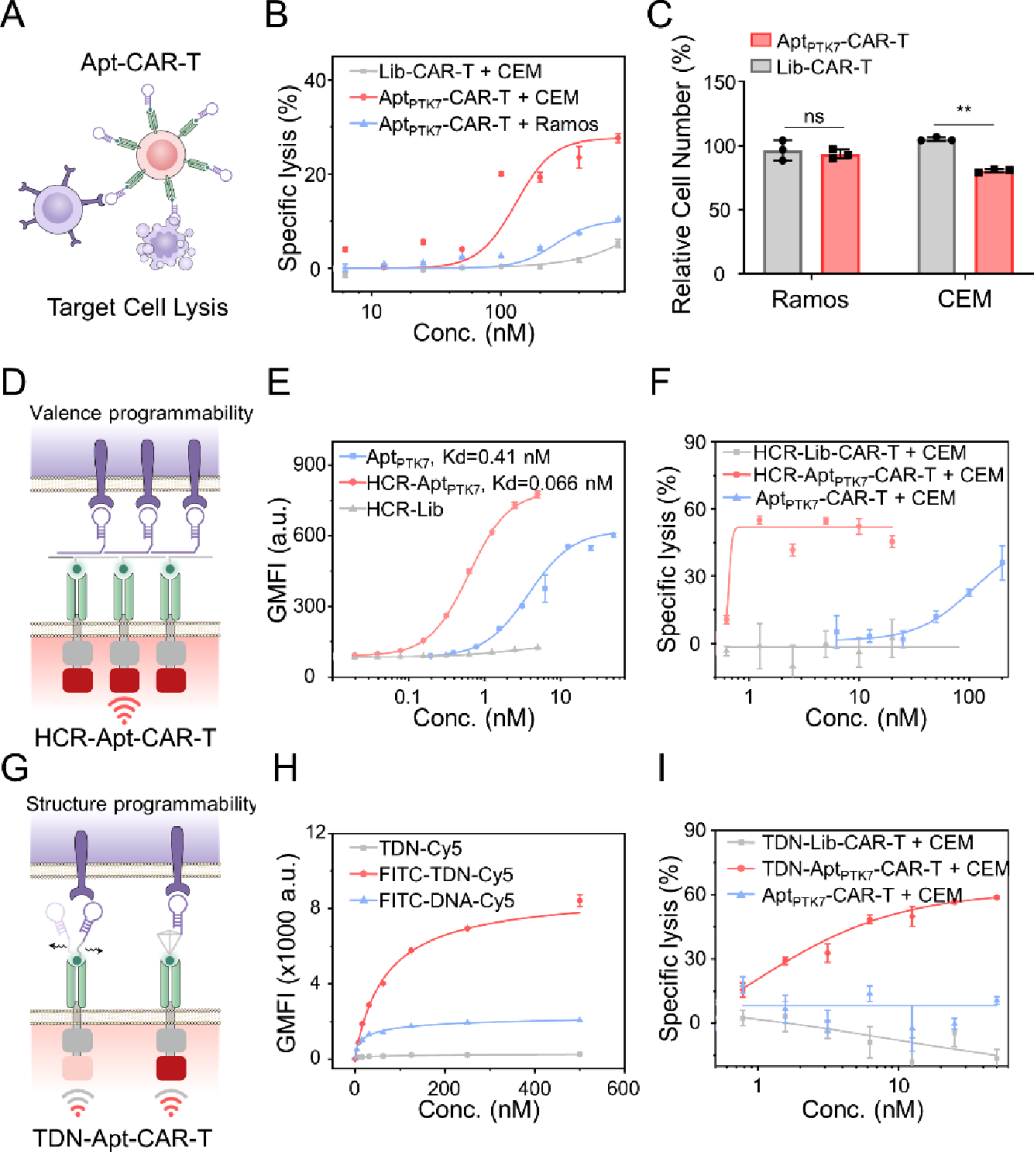
Specific target cell lysis by programmable CAR-T platform. (A) Schematic illustration of target cancer cell killing induced by Apt-CAR-T. (B) Lysis efficiency of PTK7^+^ CEM cells after coculture with Apt_PTK7_-CAR-T, or Lib-CAR-T, and PTK7^–^ Ramos after coculture with Apt_PTK7_-CAR-T for 24 h. (C) Relative cell number of CEM and Ramos after incubation with Apt_PTK7_-CAR-T (100 nM Apt_PTK7_), compared with CEM, Ramos, and Lib-CAR-T cell mixture control. Values represent relative cell number normalized to the group of mixed CAR-T, CEM, and Ramos (mixed at a cellular ratio of 10:1:1), as assayed with flow cytometry cell count beads. (D) Schematic illustration of target cancer cell killing induced by HCR-aptamer-functionalized CAR-T(HCR-Apt-CAR-T). (E) Relative FITC fluorescence intensity of CEM cells after treatment with HCR-Lib, Apt_PTK7_, and HCR-Apt_PTK7_, as assayed with flow cytometry. (F) Lysis efficiency of PTK7^+^ CEM cells after coculture with HCR-Lib-functionalized CAR-T (HCR-Lib-CAR-T) or HCR-Apt_PTK7_-functionalized CAR-T (HCR-Apt_PTK7_-CAR-T). (G) Schematic illustration of target cancer cell killing induced by TDN-aptamer-functionalized CAR-T (TDN-Apt-CAR-T). (H) Relative Cy5 fluorescence intensity of CAR-T after incubation with TDN labeled with an FITC and a Cy5 moiety (FITC-TDN-Cy5), FITC-DNA-Cy5, and TDN labeled with a Cy5 moiety (TDN-Cy5), as assayed with flow cytometry. (I) Lysis efficiency of PTK7^+^ CEM cells after coculture with TDN-Apt_PTK7_-functionalized CAR-T (TDN-Apt_PTK7_-CAR-T), TDN-Lib-functionalized CAR-T (TDN-Lib-CAR-T) or Apt_PTK7_-CAR-T. All statistical data are presented as the mean value ± S.D., *n* = 3. *P ≤ 0.05, **P ≤ 0.01, ***P ≤ 0.001, and ****P ≤ 0.0001 by two-tailed Student’ s *t* test. For all primary CAR T cell testing, three independent experiments were performed using human PBMCs collected from at least two donors.

We next tested the killing effect of Apt-CAR-T on target cancer cells (Figure 3A). As measured with lactate dehydrogenase (LDH) assay, the viability of CEM cells was significantly decreased after coculture with Apt_PTK7_-CAR-T for 24 h, reaching a half maximal effective concentration (EC_50_) at 130 nM (Figures 3B and S23). Particularly, over 27% PTK7^+^ CEM cells were eliminated by Apt_PTK7_-CAR-T, whereas only 4% PTK7^–^ Ramos cells and 0.3% PTK7^+^ CEM cells were eliminated by Apt_PTK7_-CAR-T and Lib-CAR-T, respectively. Also, we proved that Apt_PTK7_ could guide CAR-T to specifically kill PTK7^+^ CEM cells, while causing minimal impact on nontarget Ramos cells within the mixture (Figure 3C). The universality of this Apt-CAR-T design was further verified in the system of Apt_EpCAM_ against EpCAM^+^ 231 cells, but not EpCAM^–^ HeLa cells (Figure S24).

The binding avidity of CAR to target antigens, which was crucial for the effective T cell activation, relied not only on the binding affinity of individual ligands, but also on their binding valence.^35^ While the polyvalent effect could improve the antigen-binding avidity, it remains challenging to control the binding valence in antibody-based CAR design.^36^ To overcome this challenge, we utilized the programmable nature of DNA nanotechnology to develop a polyvalent CAR through aptamer-incorporated hybridization chain reaction (HCR) (Figure 3D). Successful construction of HCR-based polyvalent Apt_PTK7_ (HCR-Apt_PTK7_) and Apt_EpCAM_ (HCR-Apt_EpCAM_) was first proved with PAGE assay (Figure S25).^20,23^ Compared with the monovalent Apt, both HCR-Apt_PTK7_ and HCR-Apt_EpCAM_ exhibited enhanced target cell-binding avidity, while maintaining their recognition specificity (Figures 3E and S26). As shown in Figure 3F, based on the polyvalent effect of antigen targeting, a remarkable improvement in eradicating CEM cells was induced by HCR-Apt_PTK7_-functionalized CAR-T, especially when using a low dose of Apt (EC_50_ = 0.66 nM). In contrast, considerably lower percentage of CD69^+^CD25^+^ T cells and negligible killing effect could be detected when CAR-T was functionalized with HCR-Lib, indicating that the aptamer-based interaction with cancer cells was critical for CAR-T performance (Figure S27). Similar results were obtained in the HCR-Apt_EpCAM_ system against 231 cells (Figure S28), demonstrating the superiority of our Apt-CAR platform in leveraging polyvalency to enhance the anticancer efficacy of therapeutic T cells.

In addition to the binding avidity, the accessibility of CAR-T to target cancer cells was important in determining their efficacy.^15^ As reported, the three-dimensional tetrahedron DNA nanostructure (TDN) offers a thermodynamically favored targeting interface.^24,37^ We next tested the feasibility of using a rigid TDN as the scaffold to improve the antigen-targeting capability of Apt-functionalized CAR-T (Figure 3G). The effective synthesis of the Apt_PTK7_-tethered, FITC-labeled TDN (termed as TDN-Apt_PTK7_) was first verified (Figure S29), without compromising the binding specificity of Apt_PTK7_ to PTK7^+^ CEM cells (Figure S30). Compared with single-stranded DNA, a 3-fold enhancement on the binding efficiency between FITC-labeled DNA and anti-FITC scFv was obtained (Figure 3H). As expected, CAR-T functionalized with TDN-Apt_PTK7_ exhibited a significant increase in eliminating target CEM cells (EC_50_ = 1.71 nM), and the cell killing efficiency was 5.8-fold higher than that of the Apt_PTK7_ group (Figure 3I). Similarly, significant improvement on eradication of target cancer cells was obtained by incorporating both anti-EpCAM and anti-CD71 CAR-T with the TDN nanoscaffold (Figures S31 and S32). These results demonstrated the potential of our aptamer-functionalized CAR platform for nongenetically programming the anticancer performance of T cells.

### TME-Responsive Antigen Targeting of CAR-T

**Figure 4:**
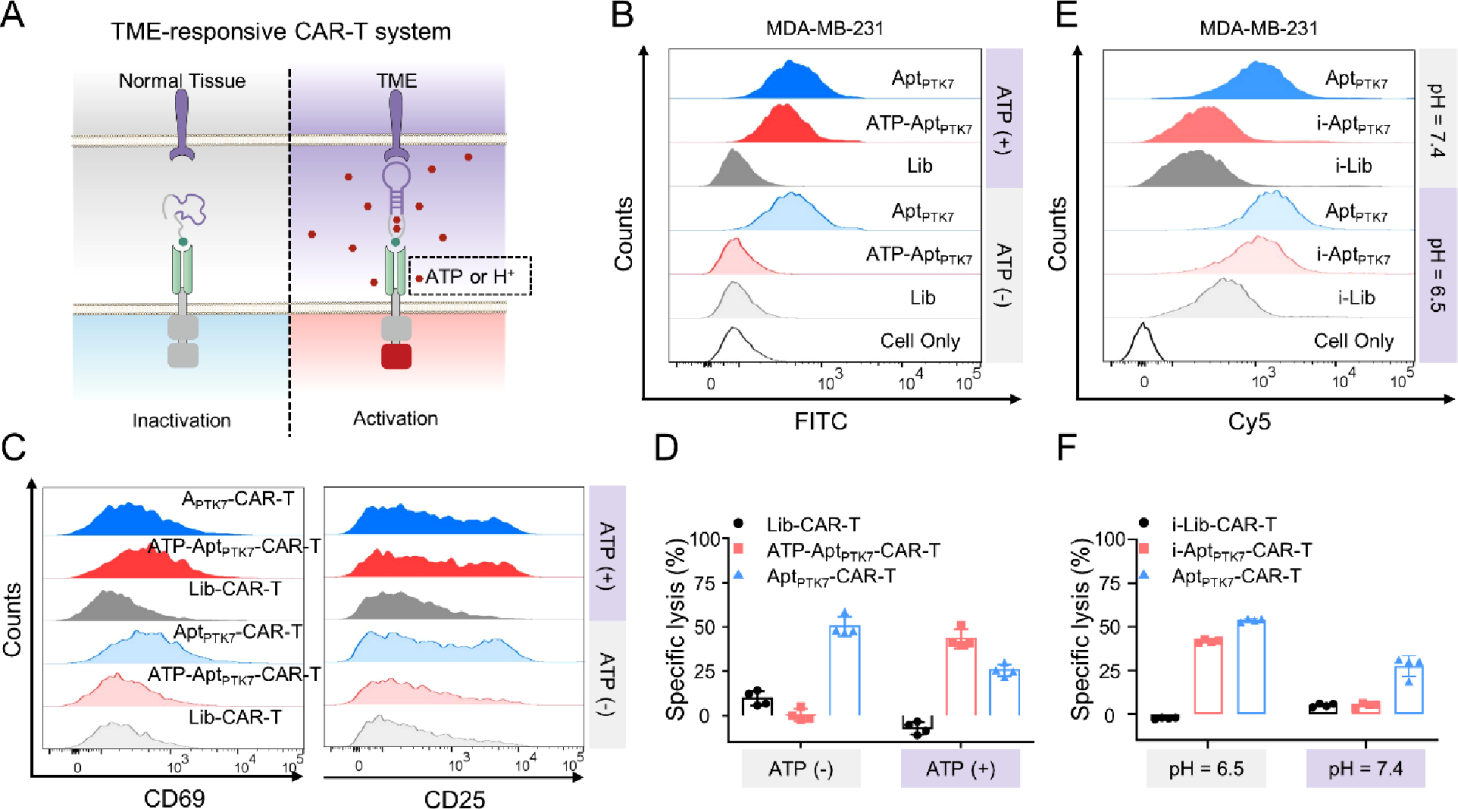
Tumor microenvironment-responsive CAR-T system. (A) Schematic illustration of tumor microenvironment (ATP or pH)-responsive target cancer cell killing of CAR-T. (B) Flow cytometry of 231 cells after incubation with 200 nM FITC-labeled Lib, ATP-Apt_PTK7_, and Apt_PTK7_ at 4 °C for 30 min in the presence (+) and absence (−) of 1 mM ATP. (C) CD69 and CD25 expression of Lib-CAR-T, ATP-Apt_PTK7_-functionalized CAR-T (ATP-Apt_PTK7_-CAR-T), or Apt_PTK7_-CAR-T after coculture with target PTK7^+^ 231 cells at 37 °C for 24 h in the presence (+) and absence (−) of 62.5 μM ATP, as assayed with flow cytometry. (D) Lysis efficiency of PTK7^+^ 231 cells after coculture with Lib-CAR-T, ATP-Apt_PTK7_-CAR-T, or Apt_PTK7_-CAR-T at 37 °C for 24 h in the presence (+) and absence (−) of 62.5 μM ATP. (E) Flow cytometry of 231 cells after incubation with 200 nM Cy5-labeled i-Lib, i-Apt_PTK7_, and Apt_PTK7_ at 4 °C at pH 6.5 or pH 7.4 for 30 min. (F) Lysis efficiency of PTK7^+^ 231 cells after coculture with i-Lib-CAR-T, i- Apt_PTK7_-CAR-T, or Apt_PTK7_-CAR-T at pH 6.5 and 7.4 for 24 h. All flow cytometric diagrams are representative data from three independent experiments. All statistical data are presented as the mean value ± S.D. (*n* = 4) *P ≤ 0.05, **P ≤ 0.01, ***P ≤ 0.001, and ****P ≤ 0.0001 by two-tailed Student’ s *t* test. For all primary CAR T cell testing, three independent experiments were performed using human PBMCs collected from at least two donors.

As tumor-associated antigens are often expressed on normal cells, the on-target off-tumor toxicity remains a significant challenge in CAR-T cell therapy.^38−40^ The hallmarks of the TME, including hypoxia,^40^ low pH,^41^ and elevated levels of adenosine triphosphate (ATP) (Figure S33),^14^ have been identified as potential targets for tumor treatments. Inspired by this, we attempted to introduce the TME responsiveness in the CAR design. As a proof-of-concept, we exploited the elevated ATP level within the TME and developed an ATP-responsive PTK7-targeting aptamer (ATP-Apt_PTK7_), in which the two ends of Apt_PTK7_ were extended with a split anti-ATP aptamer (Figure 4A). Only upon exposure to high-concentration ATP, ATP-Apt_PTK7_ could switch into a valid configuration for specific binding against PTK7^+^ cells (Figure 4B, S34, and S35). Of note, the binding of ATP-Apt_PTK7_ against PTK7^–^ Ramos cells was negligible irrespective of high or low ATP concentration in the environment, indicating the TME-responsive antigen targeting of ATP-Apt_PTK7_ (Figure S36). As indicated by the expression of CD69 and CD25, ATP-Apt_PTK7_-functionalized CAR-T (ATP-Apt_PTK7_-CAR-T) could be activated by target PTK7^+^ 231 cells in an ATP-rich environment (Figures 4C and S37), while minimal effect was observed in the absence of ATP. Also, the potency of ATP-Apt_PTK7_-CAR-T for ATP-responsive elimination of target PTK7^+^ 231 cells could be recovered only by ATP incorporation (Figure 4D). We next tested the feasibility of using another TME hallmark, low pH, to modulate the cancer-targeting specificity of Apt-CAR (Figure S38). A low pH-responsive antigen-targeting ligand was designed by extending the end of Apt_PTK7_ with a pH-switchable i-motif DNA strand,^42^ termed as i-Apt_PTK7_. The low pH-responsive binding of i-Apt_PTK7_ against PTK7^+^ 231 cells rather than PTK7^–^ Ramos cells was first verified by flow cytometry (Figures 4E, S39, and S40). As indicated by the IL-2 secretion, i-Apt_PTK7_-functionalzied CAR-T (i-Apt_PTK7_-CAR-T) could be effectively stimulated by 231 cells in a low-pH environment (Figure S41), whereas much lower IL-2 secretion was observed by coculturing i-Apt_PTK7_-CAR-T with 231 at pH 7.4 or by coculturing i-Lib-CAR-T (the fragment of Apt_PTK7_ was replaced with Lib) with 231 at pH 6.5, indicating that i-Apt_PTK7_-CAR-T could be activated by low pH-responsive antigen targeting. As expected, target cancer cells could be effectively eliminated by i-Apt_PTK7_-CAR-T only in a TME-mimic acidic environment (Figure 4F). These results suggested that the TME-responsive antigen-targeting platform provided an alternative solution for improving the tumor specificity of CAR-T cell therapy.

### In Vivo Therapeutic Effect of CAR-T

**Figure 5:**
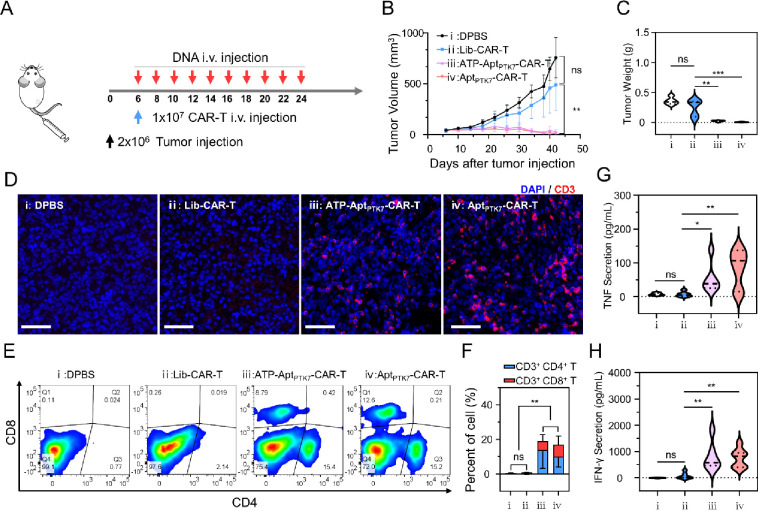
Investigating the in vivo therapeutic performance of the Apt-CAR-T system. (A) Schematic illustration of the treatment protocol for xenograft tumor mouse model. (B) Average tumor growth curves with different treatments. The tumor volume was evaluated via measuring with vernier calipers once every 4 days. (C) Statistical analysis of the weight of the tumor organs. (D) Immunofluorescence staining of tumor sections with antihuman CD3 antibody. Scale bars represent 50 μm. (E) Flow cytometry of the T cell population in PBMCs of tested mice sacrificed on day 42. (F) Statistical analysis of the percentage of CD3^+^CD4^+^ and CD3^+^CD8^+^ T cells in corresponding samples of E. (G) The serum concentration of TNF in tested mice sacrificed on day 42. (H) The serum concentration of IFN-γ in tested mice sacrificed on day 42. All statistical data are presented as the mean value ± S.D. (*n* = 5) *P ≤ 0.05, **P ≤ 0.01, ***P ≤ 0.001 and ****P ≤ 0.0001 by two-tailed Student’s *t* test.

We continued to investigate the in vivo performance of the Apt-CAR-T system (Figure 5A). To generate a human xenograft tumor mouse model, female NOD/ShiLtJGptPrkd^cem26Cd52^Il2^rgem26Cd22^/Gpt (NCG) mice were subcutaneously (*s.c*.) injected with PTK7^+^ 231 cells. Six days after tumor inoculation, the mice were randomly divided into four groups. Groups ii–iv received intravenous (*i.v*.) injections of CAR-T cells and were subsequently administrated with Lib, ATP-Apt_PTK7_, or Apt_PTK7_ every 2 days. Group i was treated with an equivalent volume of DPBS. As shown in Figure 5B, the tumor volume in Group i (DPBS) and Group ii (Lib-CAR-T) were rapidly increased, whereas the treatment of ATP-Apt_PTK7_-CAR-T (Group iii) and Apt_PTK7_-CAR-T (Group iv) significantly suppressed the tumor growth, achieving inhibition rates of 94% (*p* = 0.0089) and 98% (*p* = 0.0070), respectively. To further assess the therapeutic efficacy of these treatments, the mice were sacrificed on day 42. The results, including the weight (Figure 5C), size (Figure S42), and tissue section staining (Figure S43)) of the harvested tumor organs, all confirmed that ATP-Apt_PTK7_-CAR-T and Apt_PTK7_-CAR-T could effectively inhibit the tumor development, while no significant effect was obtained by Lib-CAR-T.

We proceeded to assess the immunological impact of CAR-T cells on tumor regression. We first examined the content and distribution of CD3^+^ T cells within the tumor tissues using immunofluorescence staining. Remarkably, in groups treated with either ATP-Apt_PTK7_-CAR-T or Apt_PTK7_-CAR-T, we observed a substantial presence of CD3^+^ T cells deep within the tumor sections (Figure 5D). In contrast, the DPBS and Lib-CAR-T groups showed minimal CD3^+^ T cell infiltration. These findings suggest that aptamer-functionalized CAR-T cells can facilitate tumor infiltration and T cell proliferation. We then evaluated the systemic immune response induced by different treatments. Flow cytometry analysis revealed a significant increase in both CD3^+^CD4^+^ and CD3^+^CD8^+^ T cells in the circulation (on day 42) in Group iii and Group iv, while only minimal levels were detected in the DPBS- and Lib-CAR-T treated groups (Figure 5E,F). Besides, compared with control Groups i and ii, treatment with ATP-Apt_PTK7_- and Apt_PTK7_-functionalized CAR-T cells effectively elevated the serum level of pro-inflammatory, TNF and IFN-γ (Figure 5G,H). These results demonstrated that the aptamer-based tumor-specific stimulation of CAR-T cells could trigger a robust systematic immune response. Notably, the serum levels of these cytokines remained within the normal range, indicating the well-tolerated nature of these treatments. Also, there were no significant differences in body weight (Figure S44) and spleen organ coefficient (Figure S45) among all tested mice. Furthermore, no apparent histological changes were observed in major organs, including the heart, liver, spleen, lung, and kidney, underscoring the biological safety of these therapeutic interventions (Figure S46). These findings collectively proved the great potential of this aptamer-functionalized CAR-T platform for combatting in vivo tumors. Importantly, the TME-responsive antigen targeting design of CAR-T (ATP-Apt_PTK7_-CAR-T) exhibited a comparable performance to that of Apt_PTK7_-CAR-T, indicating promise in tumor eradication with further enhanced specificity.

## Conclusions

Innovating CAR design is critical for addressing current limitations in CAR T-cell therapy.^43^ While many engineering strategies have been developed to optimize the performance of CAR T cells,^39−41^ potential risks and increased complexity associated with excessive genetic modification could not be overlooked.^44^ Apt-CAR, utilizing the programmable antigen recognition ability of aptamers, provided a nongenetic re-engineering platform for precise control and modulation of the T cell response against cancer cells. To the best of our knowledge, this was the first CAR-T system incorporated with aptamers. We demonstrated that CAR T cells could be directly activated through aptamer-mediated specific binding to target cancer cells. Meanwhile, aptamer-antigen binding could promote the aggregation of ligated CAR at the cellular interface and then the formation of immunological synapse, consequently leading to effective activation of T cells. As a response, activated CAR-T cells could exert specific killing effect on target cancer cells in vitro, while eliciting minimal impact on nontarget cells. In addition, the efficacy of CAR-T cell-mediated cancer elimination can be significantly augmented by leveraging the polyvalent antigen-binding effect or by enhancing antigen accessibility. We also validated the potential of this platform in the development of intelligent CAR-T cells that could selectively target antigens only in response to the TME hallmarks. Collectively, the Apt-CAR design was expected to open up new opportunities in T cell-based immunotherapy. However, further efforts are needed to improve the in vivo biostability of aptamers for clinical applications.

## Supporting Information Available

Experimental section (reagents and experimental methods); oligonucleotide sequence tables (Table S1–S6); and supporting figures (Figure S1–S46) (PDF)

## References

[ref1] June C. H.; O’Connor R. S.; et al. CAR T cell immunotherapy for human cancer. Science 2018, 359 (6382), 1361–1365. 10.1126/science.aar6711. 29567707

[ref2] Davila M. L.; Riviere I.; Wang X.; Bartido S.; Park J.; Curran K.; Chung S. S.; Stefanski J.; Borquez-Ojeda O.; Olszewska M.; Qu J.; Wasielewska T.; He Q.; Fink M.; Shinglot H.; Youssif M.; Satter M.; Wang Y.; Hosey J.; Quintanilla H.; Halton E.; Bernal Y.; Bouhassira D. C. G.; Arcila M. E.; Gonen M.; Roboz G. J.; Maslak P.; Douer D.; Frattini M. G.; Giralt S.; Sadelain M.; Brentjens R. Efficacy and Toxicity Management of 19–28z CAR T Cell Therapy in B Cell Acute Lymphoblastic Leukemia. Sci. Transl. Med. 2014, 6 (224), 224ra25 10.1126/scitranslmed.3008226. PMC4684949 24553386

[ref3] Ghassemi S.; Durgin J. S.; et al. Rapid manufacturing of non-activated potent CAR T cells. Nat. Biomed. Eng. 2022, 6 (2), 118–128. 10.1038/s41551-021-00842-6. 35190680 PMC8860360

[ref4] Zhang J.; Hu Y.; et al. Non-viral, specifically targeted CAR-T cells achieve high safety and efficacy in B-NHL. Nature 2022, 609 (7926), 369–374. 10.1038/s41586-022-05140-y. 36045296 PMC9452296

[ref5] Albelda S. M. CAR T cell therapy for patients with solid tumours: key lessons to learn and unlearn. Nat. Rev. Clin. Oncol. 2024, 21, 47 10.1038/s41571-023-00832-4. 37904019

[ref6] Yang Y.; McCloskey J. E.; et al. Bispecific CAR T Cells against EpCAM and Inducible ICAM-1 Overcome Antigen Heterogeneity and Generate Superior Antitumor Responses. Cancer Immunol. Res. 2021, 9 (10), 1158–1174. 10.1158/2326-6066.CIR-21-0062. 34341066 PMC8492509

[ref7] Wu C.-Y.; Roybal K. T.; Puchner E. M.; Onuffer J.; Lim W. A. Remote control of therapeutic T cells through a small molecule-gated chimeric receptor. Science 2015, 350 (6258), aab4077 10.1126/science.aab4077. 26405231 PMC4721629

[ref8] Huang Z.; Wu Y.; Allen M. E.; Pan Y.; Kyriakakis P.; Lu S.; Chang Y.-J.; Wang X.; Chien S.; Wang Y. Engineering light-controllable CAR T cells for cancer immunotherapy. Sci. Adv. 2020, 6 (8), 9209 10.1126/sciadv.aay9209. PMC7030928 32128416

[ref9] Stepanov A. V.; Xie J.; et al. Control of the antitumour activity and specificity of CAR T cells via organic adapters covalently tethering the CAR to tumour cells. Nat. Biomed. Eng. 2023, 10.1038/s41551-023-01102-5. 37798444

[ref10] Rafiq S.; Hackett C. S.; Brentjens R. J. Engineering strategies to overcome the current roadblocks in CAR T cell therapy. Nat. Rev. Clin. Oncol. 2020, 17 (3), 147–167. 10.1038/s41571-019-0297-y. 31848460 PMC7223338

[ref11] Jing Y.; Liu Y.; et al. Expression of chimeric antigen receptor therapy targets detected by single-cell sequencing of normal cells may contribute to off-tumor toxicity. Cancer Cell 2021, 39 (12), 1558–1559. 10.1016/j.ccell.2021.09.016. 34678153

[ref12] Tousley A. M.; Rotiroti M. C.; et al. Co-opting signalling molecules enables logic-gated control of CAR T cells. Nature 2023, 615 (7952), 507–516. 10.1038/s41586-023-05778-2. 36890224 PMC10564584

[ref13] Clauset A.; Behbakht K.; Bitler B. G. Decoding the dynamic tumor microenvironment. Sci. Adv. 2021, 7 (23), 5904 10.1126/sciadv.abi5904. PMC8177696 34088677

[ref14] Mimoto F.; Tatsumi K.; et al. Exploitation of Elevated Extracellular ATP to Specifically Direct Antibody to Tumor Microenvironment. Cell Rep. 2020, 33 (12), 108542 10.1016/j.celrep.2020.108542. 33357423

[ref15] Qi J.; Tsuji K.; et al. Chemically Programmable and Switchable CAR-T Therapy. Angew. Chem., Int. Ed. 2020, 59 (29), 12178–12185. 10.1002/anie.202005432. PMC7429910 32329959

[ref16] Hermann T.; Patel D. J. Adaptive Recognition by Nucleic Acid Aptamers. Science 2000, 287 (5454), 820–825. 10.1126/science.287.5454.820. 10657289

[ref17] Shangguan D.; Li Y.; et al. Aptamers evolved from live cells as effective molecular probes for cancer study. Proc. Natl. Acad. Sci. U. S. A. 2006, 103 (32), 11838–11843. 10.1073/pnas.0602615103. 16873550 PMC1567664

[ref18] Li S.; Jiang Q.; et al. A DNA nanorobot functions as a cancer therapeutic in response to a molecular trigger *in vivo*. Nat. Biotechnol. 2018, 36 (3), 258–264. 10.1038/nbt.4071. 29431737

[ref19] Liu C.-G.; Wang Y.; Liu; et al. Aptamer-T Cell Targeted Therapy for Tumor Treatment Using Sugar Metabolism and Click Chemistry. ACS Chem. Biol. 2020, 15 (6), 1554–1565. 10.1021/acschembio.0c00164. 32401486

[ref20] Tang R.; Fu Y.-H.; Gong B.; Fan Y.-Y.; Wang H.-H.; Huang Y.; Nie Z.; Wei P. A Chimeric Conjugate of Antibody and Programmable DNA Nanoassembly Smartly Activates T Cells for Precise Cancer Cell Targeting. Angew. Chem., Int. Ed. 2022, 134 (36), e202205902 10.1002/ange.202205902. 35751134

[ref21] Ma P.-Q.; Liu T.-X.; et al. Nano-Biohybrid DNA Engager That Reprograms the T-Cell Receptor. J. Am. Chem. Soc. 2022, 144 (49), 22458–22469. 10.1021/jacs.2c05903. 36446637

[ref22] Wang D.; Li S.; et al. Engineering a Second-Order DNA Logic-Gated Nanorobot to Sense and Release on Live Cell Membranes for Multiplexed Diagnosis and Synergistic Therapy. Angew. Chem., Int. Ed. 2021, 60 (29), 15816–15820. 10.1002/anie.202103993. 33908144

[ref23] Shi P.; Wang X.; et al. In Situ Synthesis of an Aptamer-Based Polyvalent Antibody Mimic on the Cell Surface for Enhanced Interactions between Immune and Cancer Cells. Angew. Chem., Int. Ed. 2020, 59 (29), 11892–11897. 10.1002/anie.202004206. 32307868

[ref24] Zhang J.; Lin B.; et al. DNA Nanolithography Enables a Highly Ordered Recognition Interface in a Microfluidic Chip for the Efficient Capture and Release of Circulating Tumor Cells. Angew. Chem., Int. Ed. 2020, 59 (33), 14115–14119. 10.1002/anie.202005974. 32394524

[ref25] Ma J. S. Y.; Kim J. Y.; Kazane S. A.; Choi S.-h.; Yun H. Y.; Kim M. S.; Rodgers D. T.; Pugh H. M.; Singer O.; Sun S. B.; Fonslow B. R.; Kochenderfer J. N.; Wright T. M.; Schultz P. G.; Young T. S.; Kim C. H.; Cao Y. Versatile strategy for controlling the specificity and activity of engineered T cells. Proc. Natl. Acad. Sci. U. S. A. 2016, 113 (4), E450 10.1073/pnas.1524193113. 26759368 PMC4743826

[ref26] Bloemberg D.; Nguyen T.; et al. A High-Throughput Method for Characterizing Novel Chimeric Antigen Receptors in Jurkat Cells. Mol. Ther. Methods Clin. Dev. 2020, 16, 238–254. 10.1016/j.omtm.2020.01.012. 32083149 PMC7021643

[ref27] Dustin M. L.; Cooper J. A. The immunological synapse and the actin cytoskeleton: molecular hardware for T cell signaling. Nat. Immunol. 2000, 1 (1), 23–29. 10.1038/76877. 10881170

[ref28] Song Y.; Zhu Z.; et al. Selection of DNA Aptamers against Epithelial Cell Adhesion Molecule for Cancer Cell Imaging and Circulating Tumor Cell Capture. Anal. Chem. 2013, 85 (8), 4141–4149. 10.1021/ac400366b. 23480100

[ref29] Shangguan D.; Tang Z.; et al. Optimization and Modifications of Aptamers Selected from Live Cancer Cell Lines. ChemBioChem. 2007, 8 (6), 603–606. 10.1002/cbic.200600532. 17373017

[ref30] Xiao Q.; Zhang X.; Tu L.; Cao J.; Hinrichs C. S.; Su X. Size-dependent activation of CAR-T cells. Sci. Immunol. 2022, 7 (74), 3995 10.1126/sciimmunol.abl3995. PMC9678385 35930653

[ref31] Wu X.; Zhao Z.; et al. DNA Aptamer Selected against Pancreatic Ductal Adenocarcinoma for *in vivo* Imaging and Clinical Tissue Recognition. Theranostics 2015, 5 (9), 985–994. 10.7150/thno.11938. 26155314 PMC4493536

[ref32] Scholler J.; Brady T. L.; Binder-Scholl G.; Hwang W.-T.; Plesa G.; Hege K. M.; Vogel A. N.; Kalos M.; Riley J. L.; Deeks S. G.; Mitsuyasu R. T.; Bernstein W. B.; Aronson N. E.; Levine B. L.; Bushman F. D.; June C. H. Decade-Long Safety and Function of Retroviral-Modified Chimeric Antigen Receptor T Cells. Sci. Transl. Med. 2012, 4 (132), 132ra53–132ra53. 10.1126/scitranslmed.3003761. PMC4368443 22553251

[ref33] Yang P.; Wang Y.; et al. Enhanced Safety and Antitumor Efficacy of Switchable Dual Chimeric Antigen Receptor-Engineered T Cells against Solid Tumors through a Synthetic Bifunctional PD-L1-Blocking Peptide. J. Am. Chem. Soc. 2020, 142 (44), 18874–18885. 10.1021/jacs.0c08538. 32966054

[ref34] Mukherjee M.; Mace E. M.; et al. Quantitative Imaging Approaches to Study the CAR Immunological Synapse. Mol. Ther. 2017, 25 (8), 1757–1768. 10.1016/j.ymthe.2017.06.003. 28663103 PMC5542801

[ref35] Drent E.; Themeli M.; et al. A Rational Strategy for Reducing On-Target Off-Tumor Effects of CD38-Chimeric Antigen Receptors by Affinity Optimization. Mol. Ther. 2017, 25 (8), 1946–1958. 10.1016/j.ymthe.2017.04.024. 28506593 PMC5542711

[ref36] Salzer B.; Schueller C. M.; Zajc C. U.; Peters T.; Schoeber M. A.; Kovacic B.; Buri M. C.; Lobner E.; Dushek O.; Huppa J. B.; Obinger C.; Putz E. M.; Holter W.; Traxlmayr M. W.; Lehner M. Engineering AvidCARs for combinatorial antigen recognition and reversible control of CAR function. Nat. Commun. 2020, 11 (1), 4166 10.1038/s41467-020-17970-3. 32820173 PMC7441178

[ref37] Zhao L.; Qi X.; et al. Engineering Aptamer with Enhanced Affinity by Triple Helix-Based Terminal Fixation. J. Am. Chem. Soc. 2019, 141 (44), 17493–17497. 10.1021/jacs.9b09292. 31609609

[ref38] Hernandez-Lopez R. A.; Yu W.; Cabral K. A.; et al. T cell circuits that sense antigen density with an ultrasensitive threshold. Science 2021, 371 (6534), 1166–1171. 10.1126/science.abc1855. 33632893 PMC8025675

[ref39] Fedorov V. D.; Themeli M.; Sadelain M. PD-1- and CTLA-4-Based Inhibitory Chimeric Antigen Receptors (iCARs) Divert Off-Target Immunotherapy Responses. Sci. Transl. Med. 2013, 5 (215), ra172–215ra172. 10.1126/scitranslmed.3006597. PMC4238416 24337479

[ref40] Berahovich R.; Liu X.; et al. Hypoxia Selectively Impairs CAR-T Cells. In Vitro. Cancers 2019, 11 (5), 602 10.3390/cancers11050602. 31052261 PMC6562712

[ref41] Zhang K.; Ma Y.; et al. *In Vivo* Activation of T-Cell Proliferation by Regulating Cell Surface Receptor Clustering Using a pH-Driven Interlocked DNA Nano-Spring. Nano Lett. 2022, 22 (5), 1937–1945. 10.1021/acs.nanolett.1c04562. 35225623

[ref42] Li L.; Jiang Y.; et al. Modulating Aptamer Specificity with pH-Responsive DNA Bonds. J. Am. Chem. Soc. 2018, 140 (41), 13335–13339. 10.1021/jacs.8b08047. 30212189 PMC6457906

[ref43] Labanieh L.; Majzner R. G.; Mackall C. L. Programming CAR-T cells to kill cancer. Nat. Biomed. Eng. 2018, 2 (6), 377–391. 10.1038/s41551-018-0235-9. 31011197

[ref44] Bonifant C. L.; Jackson H. J.; et al. Toxicity and management in CAR T-cell therapy. Mol. Ther. Oncolytics 2016, 3, 16011 10.1038/mto.2016.11. 27626062 PMC5008265

