# Supplementary material for: Aptamer-Based Nongenetic Reprogramming of CARs Enables Flexible Modulation of T Cell-Mediated Tumor Immunotherapy
Source: ACS Cent Sci. 2024 Mar 21;10(4):813–22. doi: 10.1021/acscentsci.3c01511 (PMC11046454; doi:10.1021/acscentsci.3c01511)
Supplement: Supplementary file 1 — oc3c01511_si_001.pdf [file oc3c01511_si_001.pdf]

Supplementary Materials for

Aptamer-Based Nongenetic Reprogramming of CARs Enables Flexible Modulation of T Cell-Mediated Tumor Immunotherapy

Qiang Zhang[1], Limei Wu[1], Yue Zhang[1], Dan Wang[2], Yingyu Sima[1], Zhimin Wang[1], Zhiwei Yin[1], Hui Wu[1], Yuting Zhuo[1], Yutong Zhang[1], Linlin Wang[1], Yong Chen[4], Yanlan Liu[1], Liping Qiu[1,2]*, Weihong Tan[2,3]*.

[1]*Molecular Science and Biomedicine Laboratory (MBL), State Key Laboratory of Chemo/Biosensing and Chemometrics, College of Chemistry and Chemical Engineering, College of Biology, Aptamer Engineering Center of Hunan Province, Hunan University, Changsha 410082, P.R. China*

[2]*The Key Laboratory of Zhejiang Province for Aptamers and Theranostics, Zhejiang Cancer Hospital, Hangzhou Institute of Medicine (HIM), Chinese Academy of Sciences, Hangzhou, Zhejiang 310022, P.R. China*

[3]*Institute of Molecular Medicine (IMM), Renji Hospital, Shanghai Jiao Tong University School of Medicine, and College of Chemistry and Chemical Engineering, Shanghai Jiao Tong University, Shanghai 200240, P.R. China*

[4]*NHC Key Laboratory of Birth Defect for Research and Prevention, Hunan Provincial Maternal and Child Health Care Hospital, Changsha 410000, P.R. China*

* *Corresponding author.*

Email: qiuliping@hnu.edu.cn;

Email: tan@hnu.edu.cn.

Content

Experimental Section 3

Reagents 3

Construction of anti-FITC CAR 3

Cell lines and cell culture 3

Isolation and transfection of primary human T cells 4

Cellular binding assay of DNA probes and antibody 5

Western blot assay 5

Construction of FITC-labeled CEM 6

Activation assay of CAR-J 6

Activation assay of CAR-T 7

Cytotoxicity assay 7

T cell proliferation assay 8

Construction of aptamer-integrated HCR product 8

Construction of aptamer-tethered DNA tetrahedron and DNA tetrahedron 9

PAGE characterization of DNA nanostructures 9

Mice feeding and treatments 9

Human cancer xenograft mouse model 10

Data replication and statistical analysis 11

Tables 12

Figures 19

Reference 66

Experimental Section

Reagents

All DNA oligonucleotides were synthesized and purified by General Biosystems (AnHui, China). DNA oligonucleotides were dissolved in TE buffer (10 mM Tris-HCl containing 1 mM EDTA, pH 8.0) and quantified according to the absorption at 260 nm with UV absorptiometry (NanoDrop™ Lite, Thermo Scientific™). Unless otherwise stated, all antibodies for flow cytometry analysis were purchased from BD Horizon™, and diluted as 1: 200 (v/v) in the binding buffer (DPBS containing 1% FBS and 5 mM $MgCl_2$) for usage.

Construction of anti-FITC CAR

Anti-FITC scFv (PDB ID: 2A9M) was designed according to previous reports and infused into second-generation lentiviral vectors with a human EF-1α promoter.[1] The sequence was optimized with codon and synthesized by General Biosystems (AnHui, China). The obtained anti-FITC CAR-T gene was inserted into pHBLV-EF1-MCS-CMV-puro vector and produced by HANBIO Biotechnology Co., Ltd. (Shanghai, China).

Cell lines and cell culture

Jurkat T (Clone E6-1), CCRF-CEM (CEM), and Ramos cell lines were purchased from ATCC and cultured in RPMI-1640 (Gibco, REF: 11875085) supplemented with 10% FBS (Gibco, REF: 10091-148) and 100 U/mL penicillin/streptomycin (Gibco, REF: 15140163) at 37 °C with 5% CO_2. MDA-MB-231 (231) and HeLa cell lines were purchased from ATCC and cultured in DMEM (Gibco, REF: 11965092) supplemented with 10% FBS and 100 U/mL penicillin/streptomycin at 37 °C in 5% CO_2. MDA-MB-231 luciferase-expressing cell line (Lu^+ 231) was obtained by transfecting MDA-MB-

231 cells with luciferase lentivirus and then screening with DMEM complete medium supplemented with 2 μg/mL puromycin (Beyotime, REF: ST551-250mg). CAR-J were cultured with RPMI-1640 complete medium supplemented with 0.5 μg/mL puromycin. The mCherry-PTK7 overexpressed K562 cell line (PTK7 overexpressed K562) was obtained by transfecting K562 cells with mCherry-PTK7 lentivirus (a gift from Jianghuai Chen at Hunan University) and then screening with 1640 complete medium supplemented with 400 μg/mL G418 (Beyotime, REF: ST081-1g).

Isolation and transfection of primary human T cells

Peripheral blood samples were collected from healthy volunteers after informed consent was approved by the Ethics Committee of Hunan Maternal and Child Health Hospital (IRB#: 202031). Primary human T cells were isolated with commercial Miltenyi MACS Cell Separation kits (CD4 Microbeads, REF: 130-045-101; CD8 Microbeads, REF: 130-045-201). To test cell purity, isolated T cells were first stained with Fixable Viability Stain 575V (BD Horizon™, REF565694) and then stained with fluorescent anti-CD3 antibody (Hu CD3 APC UCHT1, REF: 561810). Unless otherwise stated, isolated cells were stained with fluorescent anti-CD4 antibody (Hu CD4 APC-Cy7 RPA-T4, REF: 561839) and anti-CD8 antibody (Hu CD8 BV421 RPA-T8, REF: 562428) in binding buffer at 4 °C for 30 min, followed by flow cytometry analysis (Cytek DxP Athena™). T cells were cultured with AIM V medium (Gibco, REF: 12055091) supplemented with 5% heat-inactivated FBS, 100 IU/mL IL-2 (R&D Systems, REF: 202-IL), and 1 ng/mL IL-15 (R&D Systems, REF: 247-ILB).

For transfection of anti-FITC CAR, T cells were first stimulated with T Cell TransAct kits (Miltenyi, REF: 130-111-160) for 48 h, mixed with lentivirus and EnvirusTM-LV (Engreen, REF: 30001-2-1), and then centrifuged at 1200 g at 31°C for 99 min. Transfected cells were cultured from 1×10^6 to 2×10^6 cells/mL and then processed for subculture. Three days post-transfection, cells were analyzed with flow cytometry to evaluate transfection efficiency.

Cellular binding assay of DNA probes and antibody

Generally, cells (1×10^6 cells/mL) were incubated with DNA probes of different concentrations in the binding buffer at 4 °C for 30 min, adding an additional 1mM of ATP in binding and washing buffers (1640 containing 1% FBS and 5 mM $MgCl_2$) for the ATP-responsive binding experiments. After washing twice with the pre-cooled binding buffer, cells were detected with flow cytometry. To test the cellular expression of EpCAM, cells (1×10^6 cells/mL) were incubated with anti-EpCAM antibody (1:200) (abcam, REF: EPR20532-225) or Rabbit lgG (1:200) (abcam, REF: EPR25A) at 4 °C for 30 min in the binding buffer. After washing twice, cells were stained with Alexa Fluor 647 goat anti-Rabbit lgG (1:1000) (YiShan Biotech, REF: 33113ES60) at 4 °C for 30min. After removing free antibodies, cells were analyzed with flow cytometry and CLSM imaging, and accumulated data were, in turn, analyzed with FlowJo10.7.1 software and ZenBlue, respectively.

Western blot assay

CAR-J were lysed using the RIPA Lysis Buffer (Beyotime, REF: P0013B) and Protease inhibitor cocktail (Beyotime, REF: P1010) at 4 °C for 30 min. The supernatants were collected, mixed with the loading buffer (ThermoFisher, REF: B0007), and then heated at 95 °C for 10 min. The samples were loaded into 5%-12% SDS-PAGE gel, run at 60 V for 30 min and then at 120 V for 120 min. A protein leader (ThermoFisher, REF: 26617) was used as the reference. The resultant gel was transferred onto a PVDF membrane. The membrane was blocked with 5% skim milk in TBST (50 mM Tris containing 150 mM NaCl and 0.05% Tween 20) for 1 h, stained with anti-EGFP antibody (1: 1000) (Roche, REF: 11814460001) at 4 °C overnight, and finally stained with HRP Goat anti-Mouse IgG (1: 2000) (Sigma, REF: A9309-1ML) secondary antibody at room temperature for 1 h before being imaged with ChemiDoc XRS+ System (Bio-Rad).

Construction of FITC-labeled CEM

CEM cells (3×10^5 cells/mL) were suspended in RPMI-1640 complete medium containing 25 μM Ac4ManNaz (Glpbio, REF: GC60038) and allowed to incubate for 72 h. After washing with the binding buffer, cells (1×10^6 cells/mL) were incubated with 2000, 1000, 500, 250, 125, 62.5, or 0 nM FITC-DNA-DBCO at 37 °C for 60 min. To ensure an effective membrane modification, cell samples were vortexed every 15 min. After washing with DPBS, cells (termed FITC-CEM) were analyzed with flow cytometry or stored at 4 °C for further experimentation.

To evaluate the density of FITC-DNA-DBCO on the cell surface, FITC-CEM (1×10^6 cells/mL) were lysed with the RIPA lysis buffer. The supernatant was collected by centrifuge at 14000 g, 4 °C for 30 min. Fluorescence intensity of FITC in the supernatant was measured with a fluorescence spectrometer (JY-HORIBA, F-7100 FL Spectrophotometer). To quantify the concentration of FITC-DNA-DBCO, a calibration curve was obtained by measuring FITC fluorescence intensity of the RIPA solution containing the probe of given concentrations.

Activation assay of CAR-J

For the activation assay of CAR-J with FITC-DNA-conjugated CEM cells (FITC-CEM), CAR-J (1×10^5) were first stained with CellTrace Far Red (Invitrogen, REF: C34564) and then cocultured with FITC-CEM cells (2×10^5) in 200 μL RPMI-1640 complete medium at 37 °C for 24 h. For CLSM imaging, CAR-J, stained with SiR-actin Kit (Cytoskeleton, Inc. REF: CY-SC001), were mixed with FITC-DNA-CEM (cell ratio = 1: 1) and seeded at 35-mm confocal dishes for CLSM imaging.

For the activation assay of aptamer-functionalized CAR-J (Apt-CAR-J) with aptamer-based cell recognition, CAR-J (1×10^5) were first stained with CellTrace Far Red and then cocultured with specific cancer cell lines at the cellular ratio of 10:1 plus DNA probes at specific concentrations (unless otherwise statement, the final aptamer concentration was fixed at 200 nM) in 200 μL RPMI-1640 complete medium at 37 °C.

After a 24 h incubation, cells were collected, stained with anti-CD69 antibody (Hu CD69 APC FN50, REF:555533, BD Biosciences) and/or anti-CD25 antibody (Hu CD25 BV421 M-A251, REF:562442, BD Biosciences), and then analyzed with flow cytometry. Meanwhile, secreted cytokines in corresponding supernatants were collected and tested with a CBA cytokine Kit (REF:558270, 558273, 558269, BD Biosciences).

For the aptamer-mediated cell-cell assemble, mCherry-infused PTK7 overexpressed K562 ($PTK7^+$ K562) were incubated with $Apt_{PTK7\text{-}15}$ in binding buffer at 4 °C for 30 min, washed twice with washing buffer, and then mixed with SiR-Actin prestained CAR-J for 10 min at room temperature. Cells were analyzed with flow cytometry and CLSM.

Activation assay of CAR-T

CAR-T cells were incubated with specific cancer cell lines (Effector: Target = 10: 1; total cell density = 1×10^6 cells/mL) in the presence of specific DNA probes for 24 h (unless otherwise statement, the final aptamer concentration was fixed at 200 nM), adding 62.5 μM of ATP for the ATP-responsive CAR-T activation system. This was followed by staining with fluorescent anti-CD69 and anti-CD25 antibodies for flow cytometry analysis. Meanwhile, secreted cytokines in corresponding supernatants were collected and tested with a CBA cytokine kit.

Cytotoxicity assay

For adherent cells, CAR-T (9×10^5 cells/mL) were mixed with specific cell lines at a fixed cellular ratio of 10: 1 in wells of a 96-well flat-bottom plate. After adding DNA probes of different concentrations, the cell mixture was centrifuged at 300 g for 5 min and allowed to incubate for 24 h. The viability of these cell lines was evaluated with CyQUANT LDH kits (Invitrogen, REF: C20301) or bioluminescence assay (Beyotime,

REF: ST196-500 mg).

For suspension cells, CAR-T (9×10^5 cells/mL) were mixed with specific cell lines at a given ratio in wells of a 96-well U bottom plate. Aptamer probes of different concentrations were added to the cell mixture and allowed to incubate for 24 h. The viability of cancer cells was evaluated with CyQUANT LDH kits or flow cytometry-based cell count beads (BeaverBeads, REF:22306). For flow cytometry analysis, CAR-T and cancer cells were prestained with CellTrace Far Red and CellTrace Violet (Invitrogen, REF: C34557), respectively.

T cell proliferation assay

MDA-MB-231 cells were incubated with 25 μg/mL mitomycin C (Glpbio, REF: GC12353) at 37 °C for 3-5 h and then detached with 0.02% EDTA-containing DPBS. The cells were collected by centrifuge and then resuspended in AIM V complete medium deficient of cytokine. CAR-T cells (9×10^5 cells/mL) were prestained with CellTrace Far Red and then mixed with MDA-MB-231 at a cellular ratio of 2: 1. The cell mixture was transferred into wells of a 96-well flat-bottom plate. Then 100 nM DNA probes were added, and the culture medium was refreshed with 100 nM DNA probes and target cells (1×10^5 cells/mL) every 48 h and allowed to incubate for 36 h before analysis with flow cytometry.

Construction of aptamer-integrated HCR product

DNA oligonucleotides, Ini, H1, and H2 were heated at 95 °C for 10 min, immediately cooled down to 4 °C, and maintained at this temperature for 2 h. HCR products were synthesized by mixing 4 μM Ini, 40 μM H1, and 40 μM H2 in TM buffer at room temperature for 24 h. To construct HCR-Apt$_{EpCAM}$ and HCR-Apt$_{PTK7}$, H1 or H2 extended with corresponding aptamer sequences (Table S3) was mixed as described in Table S4. The concentration of HCR-Aptamer (1.82 μM) was calculated on the basis

of Ini concentration.

Construction of aptamer-tethered DNA tetrahedron and DNA tetrahedron

DNA tetrahedron (TDN) was synthesized by mixing FITC-A, B, C, and D (final concentration of each DNA strand fixed at 10 μM) in TE buffer (10 mM Tris-HCl containing 1 mM EDTA, pH 8.0) containing 12.5 mM $MgCl_2$. The DNA mixture was heated at 95 °C for 10 min and cooled down slowly at a rate of 1 °C/minute until 25°C. The aptamer-integrated DNA tetrahedron (TDN-Aptamer) was synthesized using the same protocol, except that strand A/B/C was replaced by the one extended with an aptamer sequence.

PAGE characterization of DNA nanostructures

Generally, the DNA samples (10 μL, 1 μM) were mixed with 2 μL 6× loading buffer and then loaded into wells of 5% native polyacrylamide gel electrophoresis (PAGE). The gel was run in the TAE buffer (40 mM Tris-acetate, 1 mM EDTA, 12.5 mM $MgCl_2$, pH 8.0) at 110 V for 45 min. The gel was stained with 1× Gel-Red for 15 min and then imaged with ChemiDoc XRS+ System (Bio-Rad).

Mice feeding and treatments

The ethical clearance statements. All animal experiments were conducted in accordance with institutional animal use and care regulations and approved by Animal Ethics Committee of Hunan University (No.1107271911007500). All efforts were made to reduce suffering. Female NOD/ShiLtJGptPrkd^{cem26Cd52}Il2^{rgem26Cd22}/Gpt (NCG) mice, 5−6 weeks of age, were purchased from Gempharmatech Co., Ltd (Chengdu, China). The mice were injected subcutaneously (*s.c.*) with PTK7^{+} 231 cells (2×10^6 permouse) on day 0 as the tumor cell model. When the tumor reached about 50–100 mm^3, the mice

were sacrificed, and the tumor and other main organs were harvested. The ATP concentration of obtained main organs and tumor tissues were evaluated with enhanced ATP assay kit (Beyotime, REF: S0027) according to the commercial protocol.

Human cancer xenograft mouse model

Female NCG mice, 5−6 weeks of age, were purchased from Gempharmatech Co., Ltd (Chengdu, China). The mice were s injected subcutaneously (*s.c.*) with PTK7$^+$ 231 cells (2×10^6 permouse) on day 0 as the tumor cell model, and mice were randomly divided into four group (5 mice per group). CAR-T cells (10^7 per mice) were injected intravenously (*i.v.*) in Group ii-iv on day 6. Subsequently, 100 μL DPBS or FITC-labeled Lib, ATP-Apt$_{PTK7}$ and Apt$_{PTK7}$ (100 μM) were separately infused in Group i, ii, iii, and iv through intravenous injection every other day from day 6 to 24. The tumor volume was evaluated by vernier caliper and the body weight were recorded every 4 days. The tumor volume was calculated according to the formula V=(L×W^2)/2 (L: longer diameter of tumor, W: shorter diameter of tumor). On day 42, the mice were sacrificed, and blood, main organs, and tumors were harvested for further experimental analysis. Serum from the blood sample was obtained through blood centrifugation and used for cytokine detection, the cell pellet was processed with RBC Lysis Buffer (Beyotime, REF: C3702-120mL) and then stained with Fixable Viability Stain 575V (BD Horizon™, REF565694), fluorescent anti-CD3 antibody (Hu CD3 APC UCHT1, REF: 561810), anti-CD4 antibody (Hu CD4 APC-Cy7 RPA-T4, REF: 561839) and anti-CD8 antibody (Hu CD8 BV421 RPA-T8, REF: 562428) in binding buffer at 4 °C for 30 min, followed by flow cytometry analysis (Cytek DxP Athena™). The main organs and tumors were fixed with neutrally buffered 4% paraformaldehyde, followed by hematoxylin and eosin (H&E). The PTK7 expression of tumor tissue sections were analyzed with immunohistochemistry (IHC) staining assay by servicebio biological inc (WuHan, China).

Data replication and statistical analysis

All statistical data are presented as mean values ± S.D. of three independent experiments. CLSM, PAGE, and flow cytometry results were shown by the representative data. Statistical significances were evaluated using the unpaired Student's t test, and $P < 0.05$ was considered to be statistically significant.

Tables

Table S1. Oligonucleotide Sequences of Multi-modified PolyT

Name	Sequence (5' →3')	Modification or Notes
FITC-DNA-Cy5	TTTTT TTTTT TTTTT TTTTT TTTTT TTTTT TTTTT TTTTT	5' FITC and 3' Cy5
DNA-Cy5	TTTTT TTTTT TTTTT TTTTT TTTTT TTTTT TTTTT TTTTT	3' Cy5
DNA-DBCO	TTTTT TTTTT	3' DBCO
FITC-DNA	TTTTT TTTTT	5' FITC
FITC- DNA-DBCO	TTTTT TTTTT	5' FITC and 3' DBCO

Table S2. Oligonucleotide Sequences of Aptamers

Name	Sequence (5’ →3’)	Modification
$Apt_{EPCAM-T0}$	CACTA CAGAG GTTGC GTCTG TCCCA CGTTG TCATG GGGGG TTGGC CTG	5’ FITC
$Apt_{EPCAM-T1}$	TCACT ACAGA GGTTG CGTCT GTCCC ACGTT GTCAT GGGGG GTTGG CCTG	5’ FITC
$Apt_{EPCAM-T6}$	TTTTT TCACT ACAGA GGTTG CGTCT GTCCC ACGTT GTCAT GGGGG GTTGG CCTG	5’ FITC
$Apt_{EPCAM-T11}$	TTTTT TTTTT TCACT ACAGA GGTTG CGTCT GTCCC ACGTT GTCAT GGGGG GTTGG CCTG	5’ FITC
$Apt_{PTK7-T0}$	ATCTA ACTGC TGCGC CGCCG GGAAA ATACT GTACG GTTAG A	5’ FITC
$Apt_{PTK7-T5}$	TTTTT ATCTA ACTGC TGCGC CGCCG GGAAA ATACT GTACG GTTAG A	5’ FITC
$Apt_{PTK7-T15}$	TTTTT TTTTT TTTTT ATCTA ACTGC TGCGC CGCCG GGAAA ATACT GTACG GTTAG A	5’ FITC
$Apt_{PTK7-T20}$	TTTTT TTTTT TTTTT TTTTT ATCTA ACTGC TGCGC CGCCG GGAAA ATACT GTACG GTTAG A	5’ FITC
$Apt_{CD71-T0}$	ACTCA TAGGG TTAGG GGCTG CTGGC CAGAT ACTAG ATGGT AGGGT TACTA TGAGC	5’ FITC
$Apt_{CD71-T5}$	TTTTT ACTCA TAGGG TTAGG GGCTG CTGGC CAGAT ACTAG ATGGT AGGGT TACTA TGAGC	5’ FITC
$Apt_{CD71-T10}$	TTTTT TTTTT ACTCA TAGGG TTAGG GGCTG CTGGC CAGAT ACTAG ATGGT AGGGT TACTA TGAGC	5’ FITC

Name	Sequence (5' →3')	Modification
$Apt_{CD71\text{-}T15}$	TTTTT TTTTT TTTTT ACTCA TAGGG TTAGG GGCTG CTGGC CAGAT ACTAG ATGGT AGGGT TACTA TGAGC	5' FITC
$Apt_{CD71\text{-}T20}$	TTTTT TTTTT TTTTT TTTTT ACTCA TAGGG TTAGG GGCTG CTGGC CAGAT ACTAG ATGGT AGGGT TACTA TGAGC	5' FITC
Lib	TTTTT TTTGC AGTTG ATCCT TTTTT TTTTG GATAC CCTGG TTTGG ATACC CTGG	5' FITC
ATP- Apt_{PTK7}	CTGGGGGAGTATACTGCTGCGCCGCCG GGAAAATACTGTACGGTTGCGGAGGAA GG	5' FITC
i-Lib	CCCCCTTTTCCCCCATAACTGCTGCGCC GCCGGGAAAATACTGTACGGTTAACCC CCTTTTCCCCCTTTTT	3' FITC or Cy5
i-Apt_{PTK7}	CCCCCTTTTCCCCCAGCTGCTCGCCGTA ACTCCGATACGGAAGGTTATGTACAGA ACCCCCTTTTCCCCC	3' FITC or Cy5

Note: the aptamer sequence was labeled in black, and the T linker was labeled in blue. $Apt_{EPCAM\text{-}T6}$, $Apt_{PTK7\text{-}T15}$, and $Apt_{CD71\text{-}T20}$ was chosen as the optimal design and shortly termed as Apt_{EPCAM}, Apt_{PTK7}, and Apt_{CD71} respectively, in subsequent studies.

Table S3. Oligonucleotide Sequences of Polyvalent Aptamers

Name	Sequence (5' →3')	Modification
Ini	CCCAG GTTCT CTTTT TTT	
H1	AAAAA GAGAA CCTGG GTACG ATGCC CAGGT TC	5' FITC
H2	CCCAG GTTCT CTTTT TGAAC CTGGG CATCG TA	5' FITC
H1-Apt$_{PTK7}$	ATCTA ACTGC TGCGC CGCCG GGAAA ATACT GTACG GTTAG ATTTT TTTTT TTTTT TAAAA AGAGA ACCTG GGTAC GATGC CCAGG TTC	
H2-Apt$_{EpCAM}$	CCCAG GTTCT CTTTT TGAAC CTGGG CATCG TATTT TTTCA CTACA GAGGT TGCGT CTGTC CCACG TTGTC ATGGG GGGTT GGCCT G	
H2-Lib	CCCAG GTTCT CTTTT TGAAC CTGGG CATCG TATTT TTTTT	

Note: the aptamer sequence was labeled in blue, and the HCR sequence and linker were labeled in black.

Table S4. Sequence used for Synthesis of Polyvalent Aptamers

Name	Sequence
HCR-Lib	Ini + H1 + H2-Lib
HCR-Apt_{EpCAM}	Ini + H1 + H2-Apt_{EpCAM}
HCR-Apt_{PTK7}	Ini + H1- Apt_{PTK7} + H2

Table S5. Oligonucleotide Sequences of TDN and TDN-aptamers

**Name**	**Sequence (5' →3')**	Modification
A	ACATTC CTAAG TCTGA AACAT TACAG CTTGC TACAC GAGAA GAGCC GCCAT AGTA	
B	TTTAT CACCA GGCAG TTGAC AGTGT AGCAA GCTGT AATAG ATGCG AGGGT CCAA TAC	
C	TTTCA ACTGC CTGGT GATAA AACGA CACTA CGTGG GAATC TACTA TGGCG GCTCT TC	
D	TTTTC AGACT TAGGA ATGTG CTTCC CACGT AGTGT CGTTT GTATT GGACC CTCGC AT	
A-Cy5	ACATT CCTAA GTCTG AAACA TTACA GCTTG CTACA CGAGA AGAGC CGCCA TAGTA	5' Cy5
A-Apt$_{EpCAM}$	CACTA CAGAG GTTGC GTCTG TCCCA CGTTG TCATG GGGGG TTGGC CTTTT TTACA TTCCT AAGTC TGAAA CATTA CAGCT TGCTA CACGA GAAGA GCCGC CATAG TA	
B-Apt$_{PTK7}$	ATCTA ACTGC TGCGC CGCCG GGAAA ATACT GTACG GTTAG ATTTA TCACC AGGCA GTTGA CAGTG TAGCA AGCTG TAATA GATGC GAGGG TCCAA TAC	
C-Apt$_{CD71}$	ACTCA TAGGG TTAGG GGCTG CTGGC CAGAT ACTAG ATGGT AGGGT TACTA TGAGC TTTTT TTCAA CTGCC TGGTG ATAAA ACGAC ACTAC GTGGG AATCT ACTAT GGCGG CTCTTC	
FITC-D	TTTTC AGACT TAGGA ATGTG CTTCC CACGT AGTGT CGTTT GTATT GGACC CTCGC AT	5' FITC

Note: aptamer sequence was labeled in blue, and the TDN framework sequence and linker were labeled in black.

Table S6. Sequence used for TDN-aptamer Synthesis

Name	Sequence
FITC-TDN-Cy5	A-Cy5 + B + C + FITC-D
TDN-Cy5	A-Cy5 + B + C + D
TDN-Apt_{EpCAM}	A-Apt_{EpCAM} + B + C + FITC-D
TDN-Apt_{PTK7}	A + B-Apt_{PTK7} + C + FITC-D
TDN-Apt_{CD71}	A + B + C-Apt_{CD71} + FITC-D
TDN-Lib	A + B + C + FITC-D

Figures

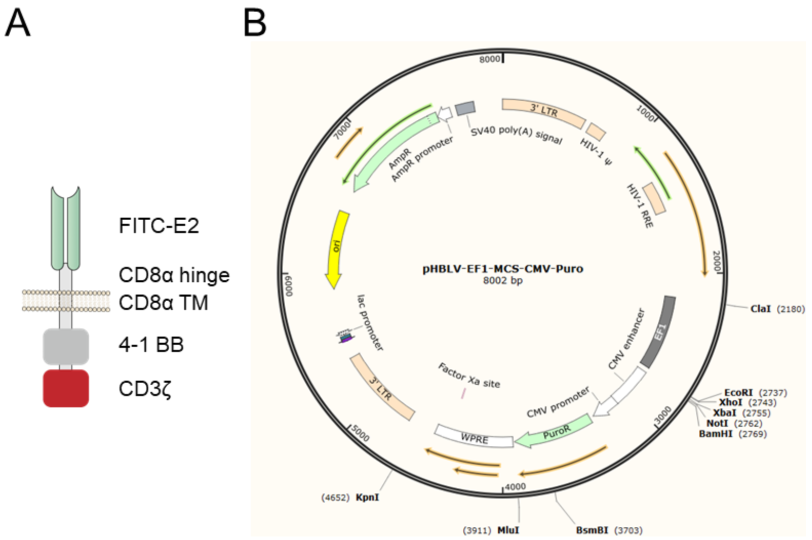

Figure S1. (**A**) Structure of anti-FITC CAR. (**B**) Lentiviral vector plasmid used in this work (provided by HANBIO Biotechnology Co., Ltd. (Shanghai, China)).

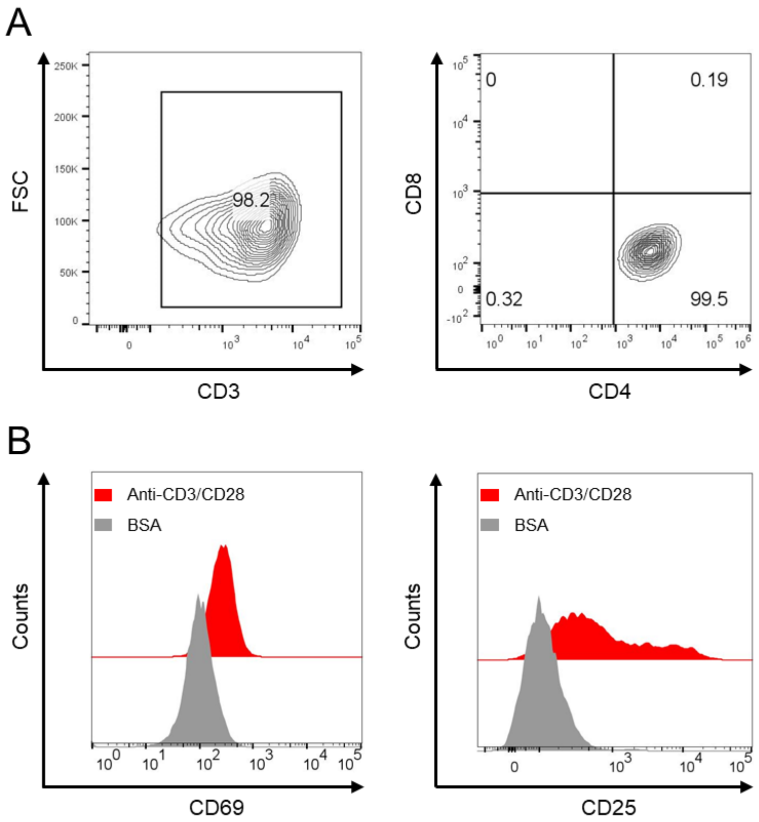

Figure S2. (**A**) Flow cytometry analysis of Jurkat T cells stained with fluorescent antibodies of CD3 (APC), CD4 (APC-Cy7) and CD8 (BV421). (**B**) Flow cytometry analysis. Jurkat T cells were incubated in wells of a 96 well-plate pretreated with anti-CD3 (1 μg/mL) and anti-CD28 (5 μg/mL) at 37 °C for 24 h and then stained with fluorescent antibodies of CD69 (PE) and CD25 (PE-Cy7). Jurkat T cells incubated in bovine serum albumin (BSA)-pretreated plate were used as a negative activation control. Data are representative data from three independent experiments.

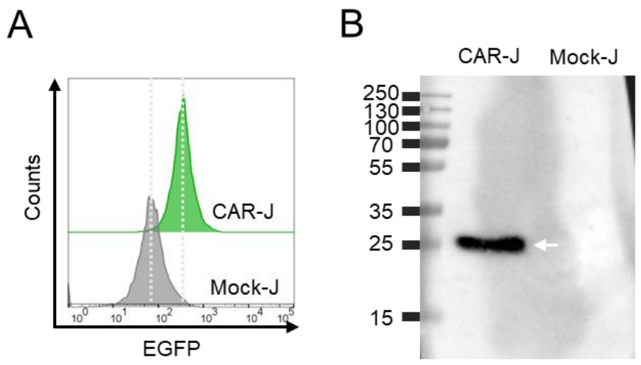

Figure S3. (**A**) Flow cytometry analysis of Jurkat T cells with (CAR-J) or without (Mock-J) transfection of EGFP-fused anti-FITC CAR. Data are representative data from three independent experiments. (**B**) Western blotting analysis of corresponding cellular lysate stained with anti-EGFP antibody. According to the protein ladder (15 kDa-250 kDa), the band indicated by the white arrowhead represented the EGFP fragment (~27 kDa), rather than the whole EGFP-fused anti-FITC CAR (~96 kDa). Data are representative data from two independent experiments.

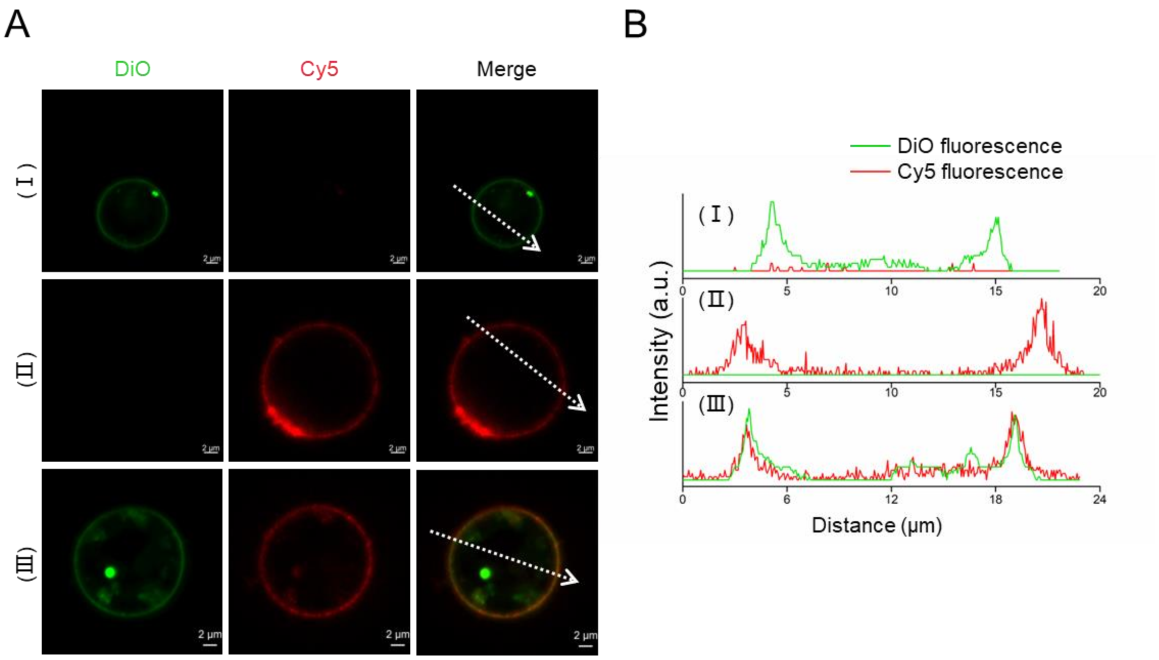

Figure S4. (**A**) CLSM imaging of CAR-J cells stained with membrane dye DiO (I), FITC-DNA-Cy5 (II) or DiO plus FITC-DNA-Cy5 (III). Scale bars represent 2 μm. (**B**) Fluorescence profile of DiO (green) and Cy5 (red) along corresponding white dotted line in merge images of A.

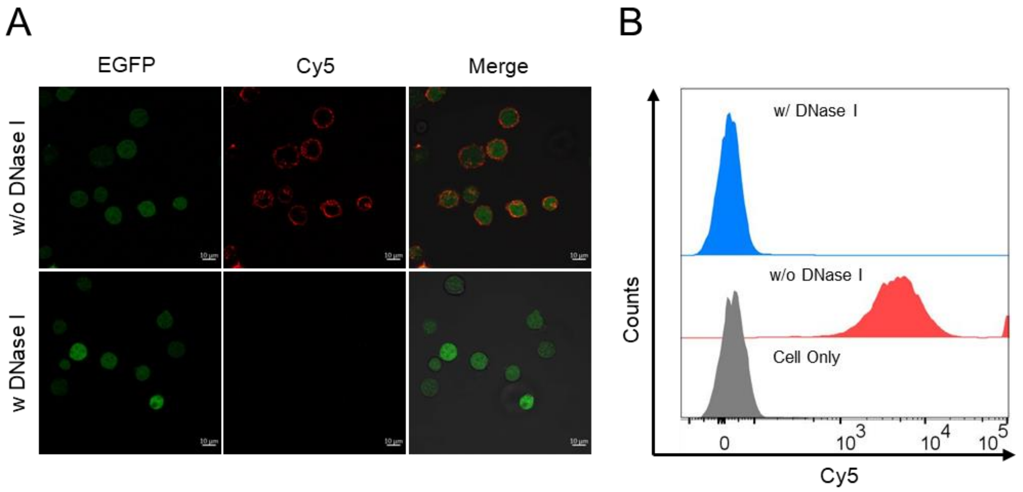

Figure S5. (**A**) CLSM imaging of FITC-DNA-Cy5-coupled CAR-J cells with or without treatment of 5 U/mL DNase I in binding buffer at 37 °C for 15 min. Scale bars represent 10 μm. (**B**) Flow cytometry analysis of FITC-DNA-Cy5-coupled CAR-J cells with or without treatment of 5 U/mL DNase I in binding buffer at 37 °C for 15 min. Data are representative data from three independent experiments.

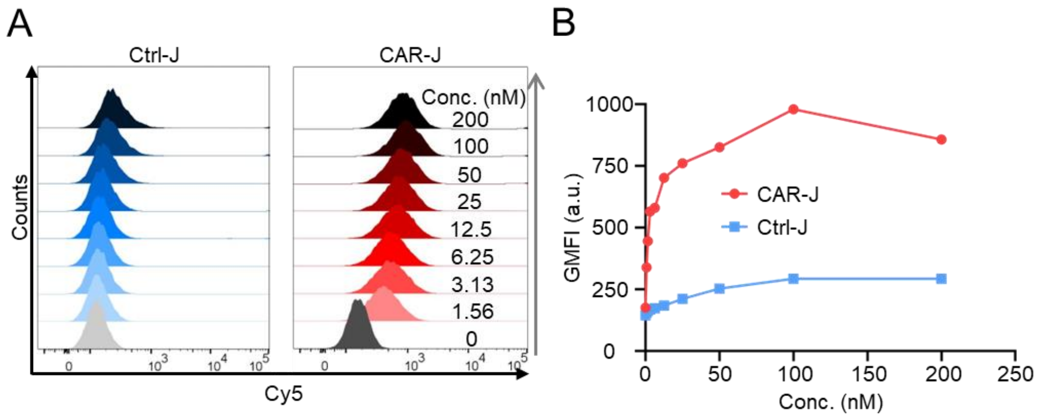

Figure S6. (**A**) Flow cytometry analysis of CAR-J or control Jurkat cells transfected with blank lentiviral vector (Ctrl-J) after incubation with FITC-DNA-Cy5 of different concentrations in binding buffer at 4 °C for 30 min. (**B**) Geometric mean fluorescence intensity (GMFI) of Cy5 in corresponding samples of A.

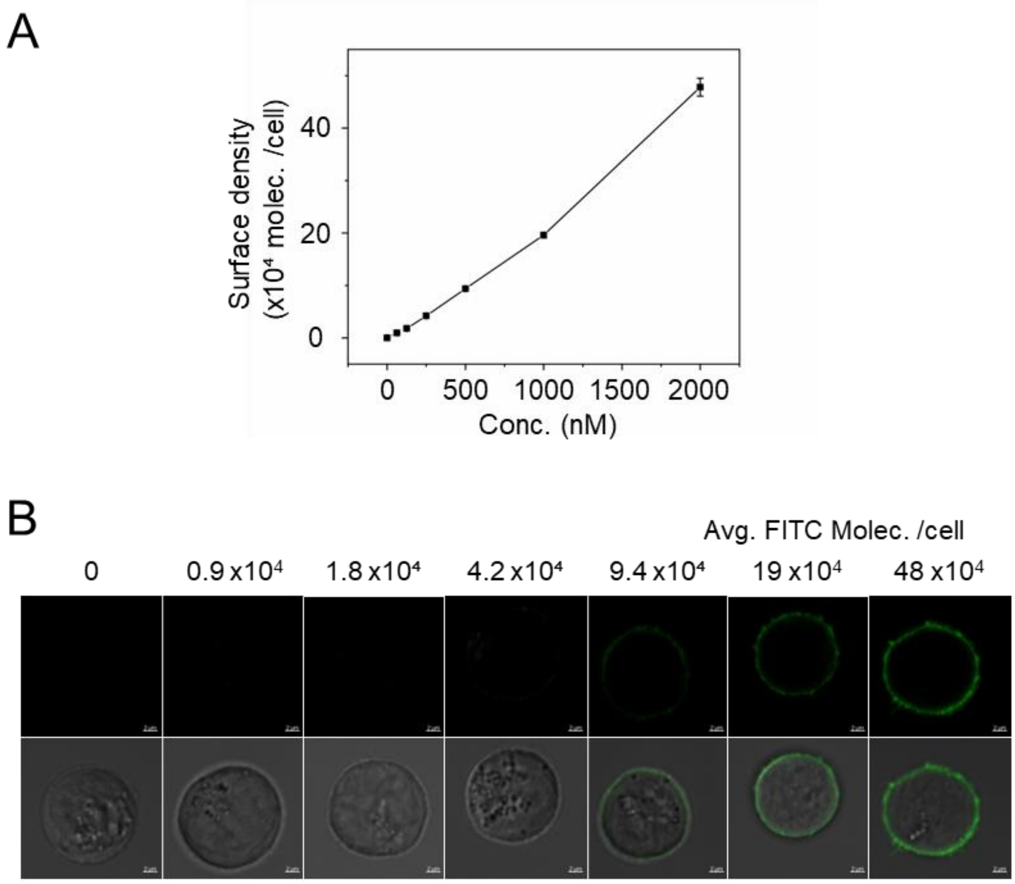

Figure S7. (**A**) Flow cytometry analysis of Ac4ManNAz-pretreated CEM cells after incubation with FITC-DNA-DBCO of different concentrations in the binding buffer at 37 ^{o}C for 1 h in corresponding samples of Figure 1E. The cell surface density of the DNA ligand was calculated according to the FITC fluorescence of the cell lysates. Data are presented as the mean value ± S.D., n = 3. (**B**) CLSM imaging of CEM cells decorated with FITC-DNA of different surface density. Scale bars represent 2 μm.

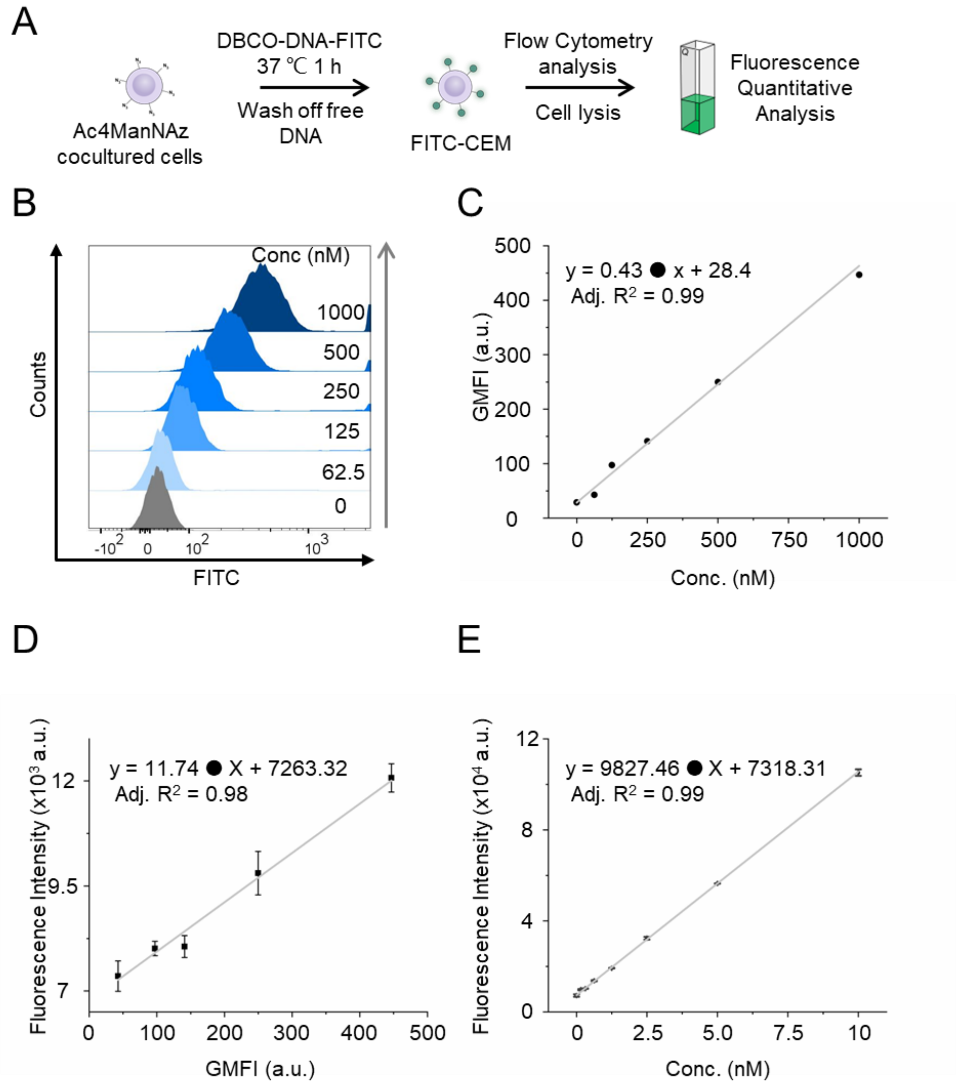

Figure S8. (**A**) Schematic illustration of constructing CEM cells conjugated with FITC-DNA (FITC-CEM) and analysis of FITC molecular density through cell lysis supernatant fluorescence intensity. (**B**) Flow cytometry analysis of CEM cells processed with Ac4ManNAz and then incubated with FITC-DNA-DBCO of different concentrations (0-1000 nM) in binding buffer at 37 ^{o}C for 1 h. Data are representative data from three independent experiments. (**C**) GMFI of FITC in corresponding samples of B (n = 3). (**D**) FITC fluorescence intensity of the cell lysate supernatant of corresponding sample of b, as assayed with fluorescence spectroscopy (n = 3). (**E**) FITC fluorescence intensity of FITC-DNA-DBCO at different concentrations in RIPA lysis buffer (n = 3).

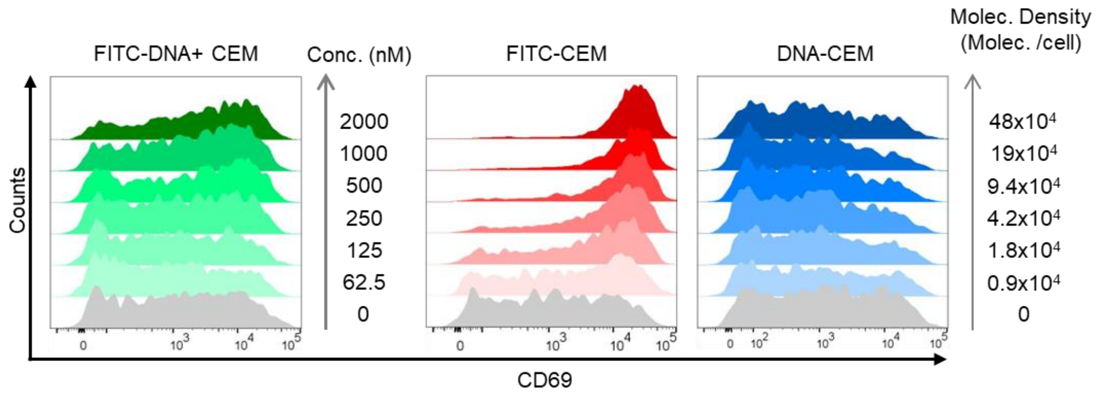

Figure S9. Flow cytometry analysis of the CD69 expression. CAR-J were cocultured with CEM cells plus free FITC-DNA of different concentrations, CEM cells conjugated with DNA of different surface density (DNA-CEM), or CEM cells conjugated with FITC-DNA of different surface density (FITC-CEM) at 37 ^{o}C for 24 h, and then stained with APC-conjugated anti-CD69 antibody, before analysis with flow cytometry. The molar ratio between CAR-J and CEM was fixed at 2:1. Data are representative data from three independent experiments.

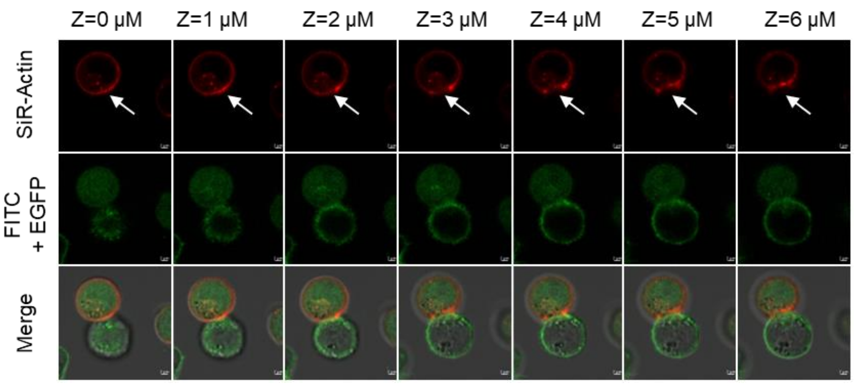

Figure S10. CLSM z-stack images of CAR-J (stained with SiR-Actin) with interaction of FITC-CEM. Scale bars represent 2 μm. White arrowhead points to the immunological synapse (IS), revealing the recruitment and enrichment of actin around the periphery of IS.

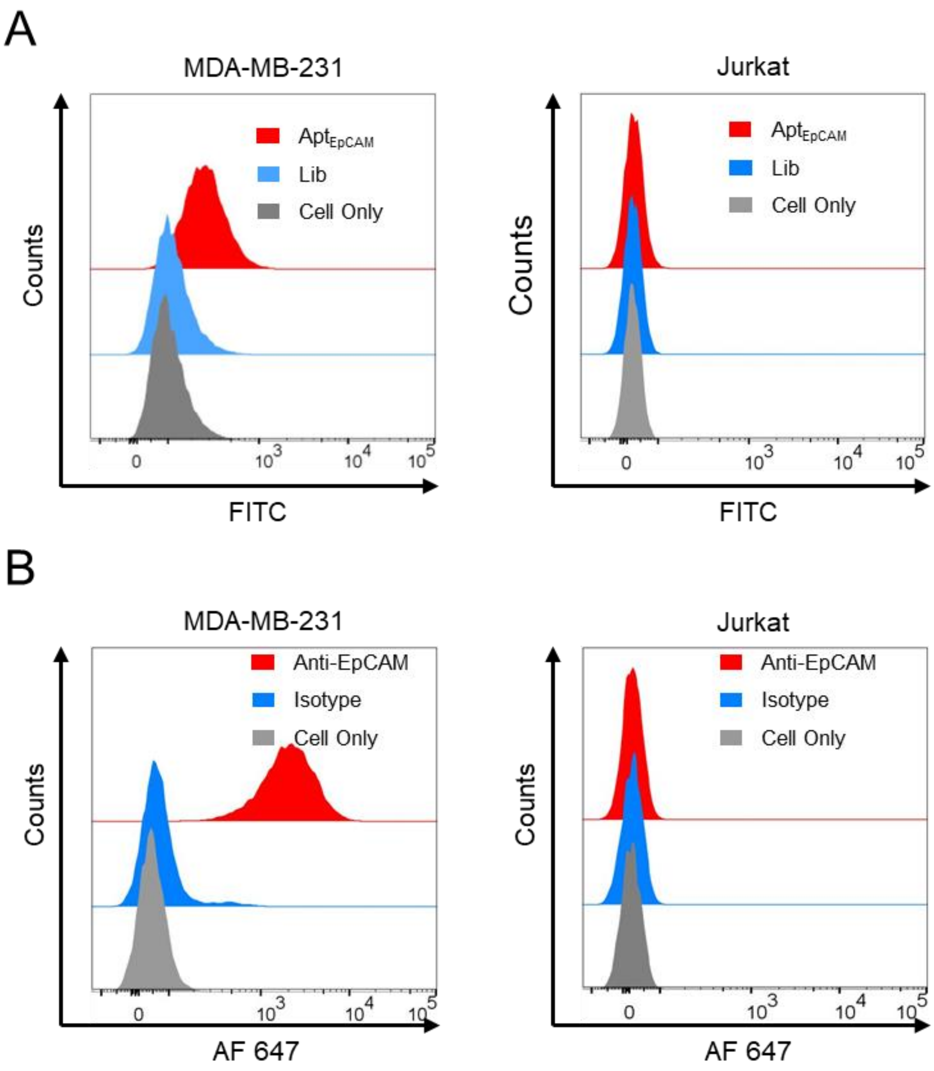

Figure S11. (**A**) Flow cytometry analysis of MDA-MB-231 and Jurkat cells incubated with 200 nM Lib or Apt_{EpCAM} in binding buffer at 4 ^{o}C for 30 min. (**B**) Flow cytometry analysis of MDA-MB-231 and Jurkat cells incubated with anti-EpCAM antibody or isotype control (1:200) in binding buffer at 4 ^{o}C for 30 min. Flow cytometry data are representative data from three independent experiments.

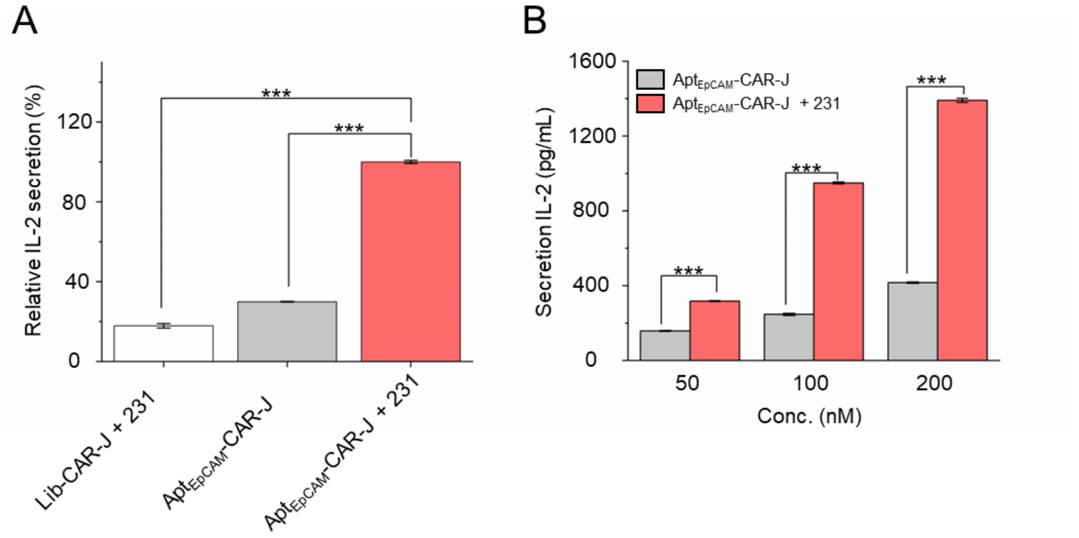

Figure S12. (**A**) Relative IL-2 secretion of Lib-functionalized CAR-J (Lib-CAR-J) after coculture with MDA-MB-231 cells (231) at 37 °C for 24 h, or Apt$_{EpCAM}$-functionalized CAR-J (Apt$_{EpCAM}$-CAR-J) after coculture with or without 231cells at 37 °C for 24 h, as assayed with BD CBA cytokine kit. (**B**) IL-2 secretion of CAR-J functionalized with Apt$_{EpCAM}$ of different concentrations after incubation with (red) or without (gray) target 231 cells at 37 °C for 24 h. All statistical data are presented as the mean value ± S.D., n = 3. *P ≤ 0.05, **P ≤ 0.01, and ***P ≤ 0.001 and ****P ≤ 0.0001 by two-tailed Student's *t*-test.

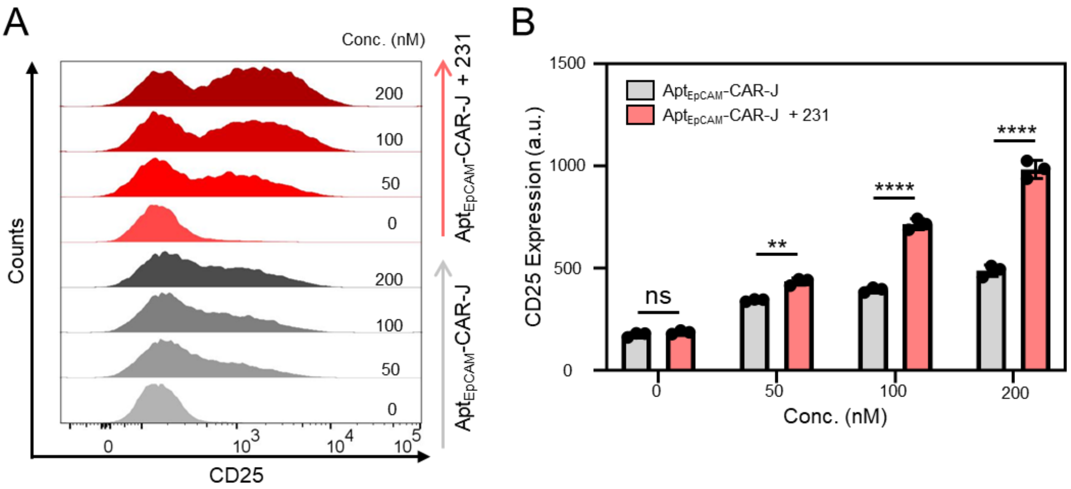

Figure S13. (**A**) Flow cytometry analysis of the CD25 expression. CAR-J were functionalized with Apt_{EpCAM} of different concentrations, cocultured with or without target 231 cells at 37 °C for 24 h, and then stained with BV421-conjugated anti-CD25 antibody. (**B**) Relative CD25 expression in corresponding samples of A. Data are presented as the mean value ± S.D., n = 3. *P ≤ 0.05, **P ≤ 0.01, ***P ≤ 0.001, and ****P ≤ 0.0001 by two-tailed Student's t-test.

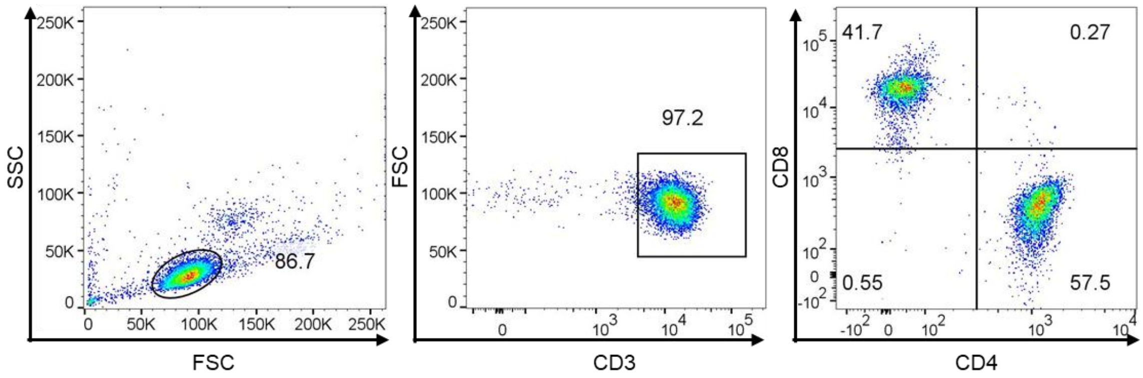

Figure S14. Flow cytometry analysis of primary T cells isolated from PBMCs and then stained with anti-CD3 (APC), anti-CD4 (APC-Cy7), and anti-CD8 (BV421) at 4 ^{o}C for 30 min.

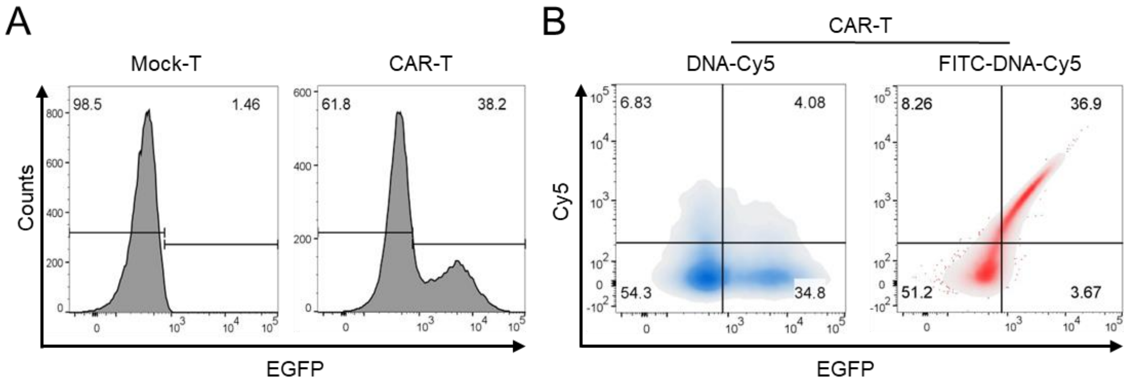

Figure S15. (**A**) Flow cytometry analysis of primary T cells transfected with (CAR-T) or without (Mock-T) EGFP-fused anti-FITC CAR. (**B**) Flow cytometry analysis of CAR-T stained with 50 nM DNA-Cy5 or FITC-DNA-Cy5 at 4 ℃ for 30 min. Flow cytometry data are representative data from three independent experiments.

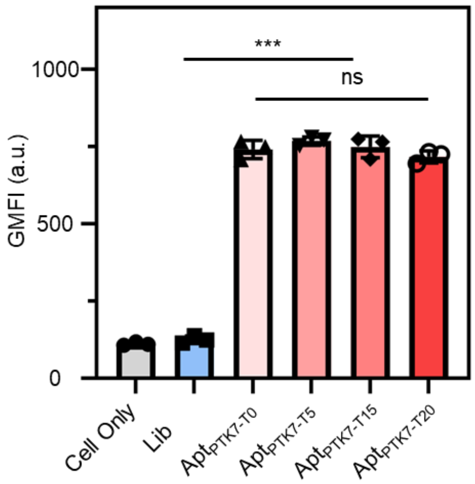

Figure S16. Flow cytometry of CEM cells after incubation with 200 nM FITC-labeled Lib, Apt$_{PTK7-T0}$, Apt$_{PTK7-T5}$, Apt$_{PTK7-T15}$, or Apt$_{PTK7-T20}$ in the binding buffer at 4 ^{o}C for 30 min. This result represents the statistical analysis of the flow cytometry data shown in Figure 2B. Data are presented as the mean value ± S.D., n = 3. *P ≤ 0.05, **P ≤ 0.01, ***P ≤ 0.001, and ****P ≤ 0.0001 by two-tailed Student's t-test.

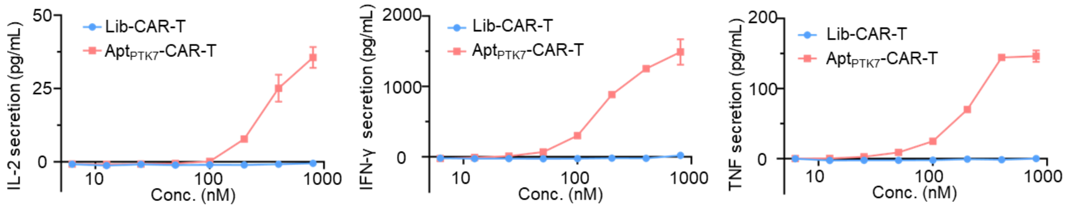

Figure S17. IL-2, IFN-γ and TNF secretion of $PTK7^{+}$ CEM cells after coculture with Apt_{PTK7}-CAR-T, or Lib-CAR-T for 24 h, as assayed with flow cytometry. Data are presented as the mean value ± S.D., n = 3.

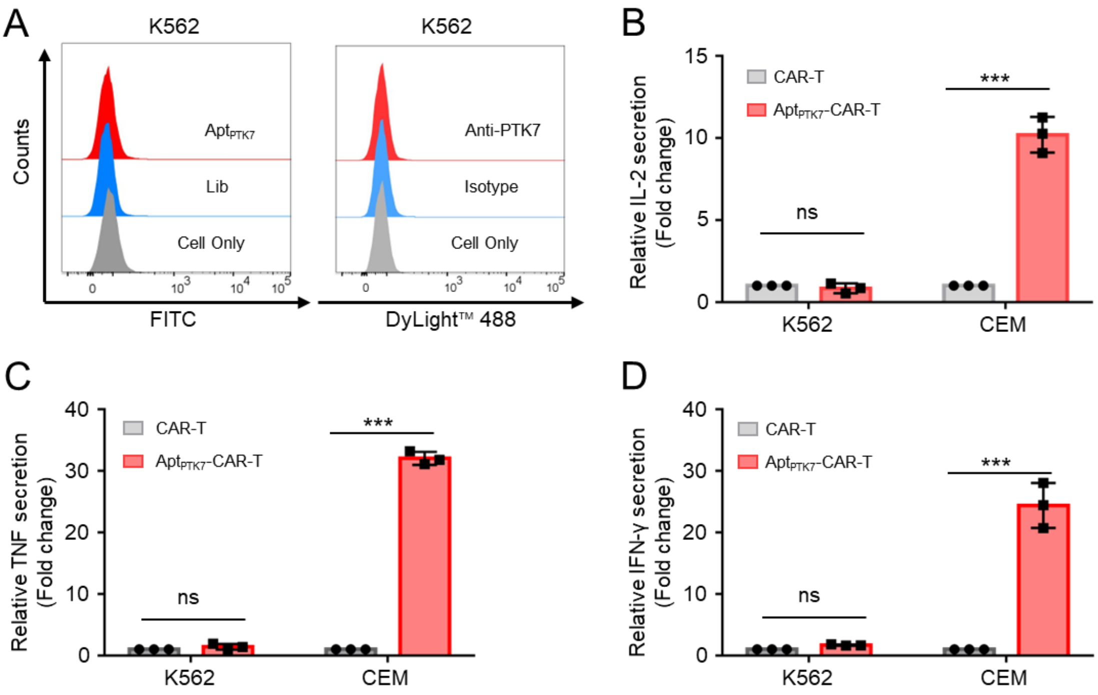

Figure S18. (**A**) Flow cytometry analysis of K562 cells incubated with 200 nM Lib, Apt$_{PTK7}$, Isotype control (1:200) and anti-PTK7 antibody (1:200) in binding buffer at 4 ^{o}C for 30 min. Flow cytometry data are representative data from three independent experiments. (**B**) Relative IL-2, (**C**) TNF, and (**D**) INF-γ secretion fold change of Apt$_{PTK7}$-functionalized CAR-T (Apt$_{PTK7}$-CAR-T) cocultured with PTK7^{+} CEM cells or PTK7^{-} K562 cells at 37 ^{o}C for 24 h, compared with CAR-T cocultured with PTK7^{+} CEM cells or PTK7^{-} K562 cells. Values represent relative cytokine secretion normalized to the CAR-T coculture with CEM/K562. The molar ratio between CAR-T and CEM/K562 cells was fixed at 10: 1. All statistical data are presented as the mean value ± S.D., n = 3. *P ≤ 0.05, **P ≤ 0.01, ***P ≤ 0.001, and ****P ≤ 0.0001 by two-tailed Student's *t*-test.

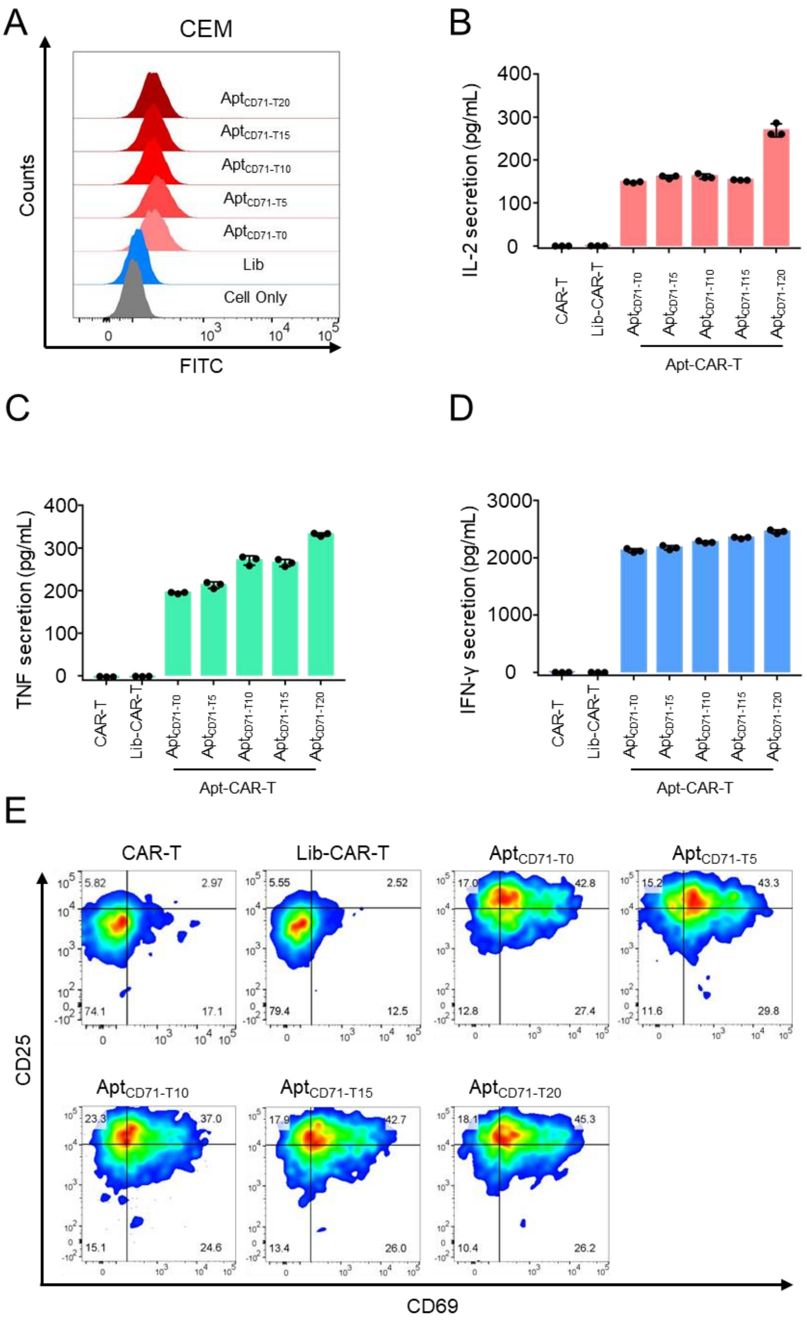

Figure S19. (**A**) Flow cytometry analysis of CEM cells incubated with 200 nM Lib, Apt$_{CD71-T0}$, Apt$_{CD71-T5}$, Apt$_{CD71-T10}$, Apt$_{CD71-T15}$ and Apt$_{CD71-T20}$ in binding buffer at 4 °C for 30 min. (**B**) IL-2, (**C**) TNF, and (**D**) INF-γ secretion of CAR-T, Lib-CAR-T, and CAR-T functionalized with Apt$_{CD71-T0}$, Apt$_{CD71-T5}$, Apt$_{CD71-T10}$, Apt$_{CD71-T15}$, or Apt$_{CD71-T20}$ after coculture with CD71$^+$ CEM cells at 37 °C for 24 h. (**E**) CD69 (APC) and CD25 (BV421) expression of the corresponding samples of B, as assayed with flow cytometry. Molar ratio between CAR-T and CEM cells was fixed at 10: 1. In all

subsequent studies, $Apt_{CD71\text{-}T20}$ and $Apt_{CD71\text{-}T20}$-functionalized CAR-T was chosen as the optimal design and shortly termed as Apt_{CD71} and Apt_{CD71}-CAR-T, respectively. All flow cytometry data are representative data from three independent experiments. All statistical data are presented as the mean value ± S.D., n = 3.

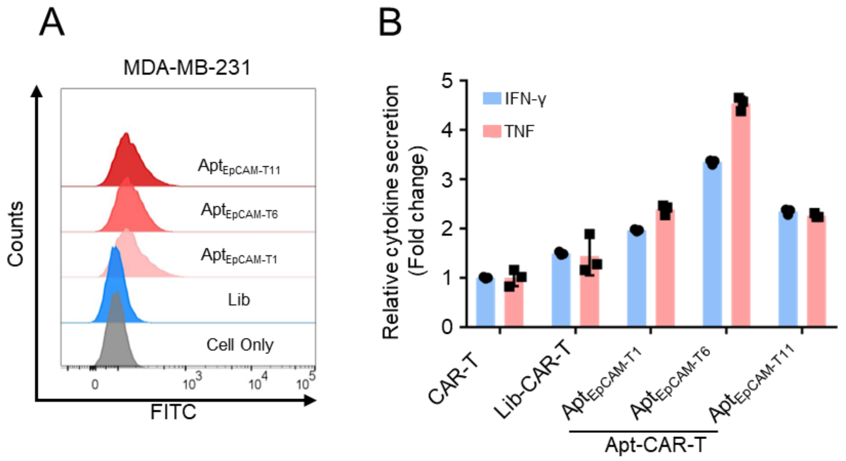

Figure S20. (**A**) Flow cytometry analysis of MDA-MB-231 cells incubated with 200 nM Lib, $Apt_{EpCAM-T1}$, $Apt_{EpCAM-T6}$, or $Apt_{EpCAM-T11}$ at 4 °C for 30 min. Data are representative data from three independent experiments. (**B**) Relative INF-γ and TNF cytokine secretion of CAR-T, Lib-CAR-T, and CAR-T functionalized with $Apt_{EpCAM-T1}$, $Apt_{EpCAM-T6}$, or $Apt_{EpCAM-T11}$ after coculture with 231 cells at 37 °C for 24 h. Values represent relative cytokine secretion normalized to CAR-T coculture with 231. The concentration of functionalized Lib/Aptamer was 200 nM, and the molar ratio between CAR-T and 231 cells was fixed at 10: 1. In all subsequent studies, $Apt_{EpCAM-T6}$ and $Apt_{EpCAM-T6}$-functionalized CAR-T was chosen as the optimal design and shortly termed as Apt_{EpCAM} and Apt_{EpCAM}-CAR-T, respectively. Statistical data are presented as the mean value ± S.D., n = 3.

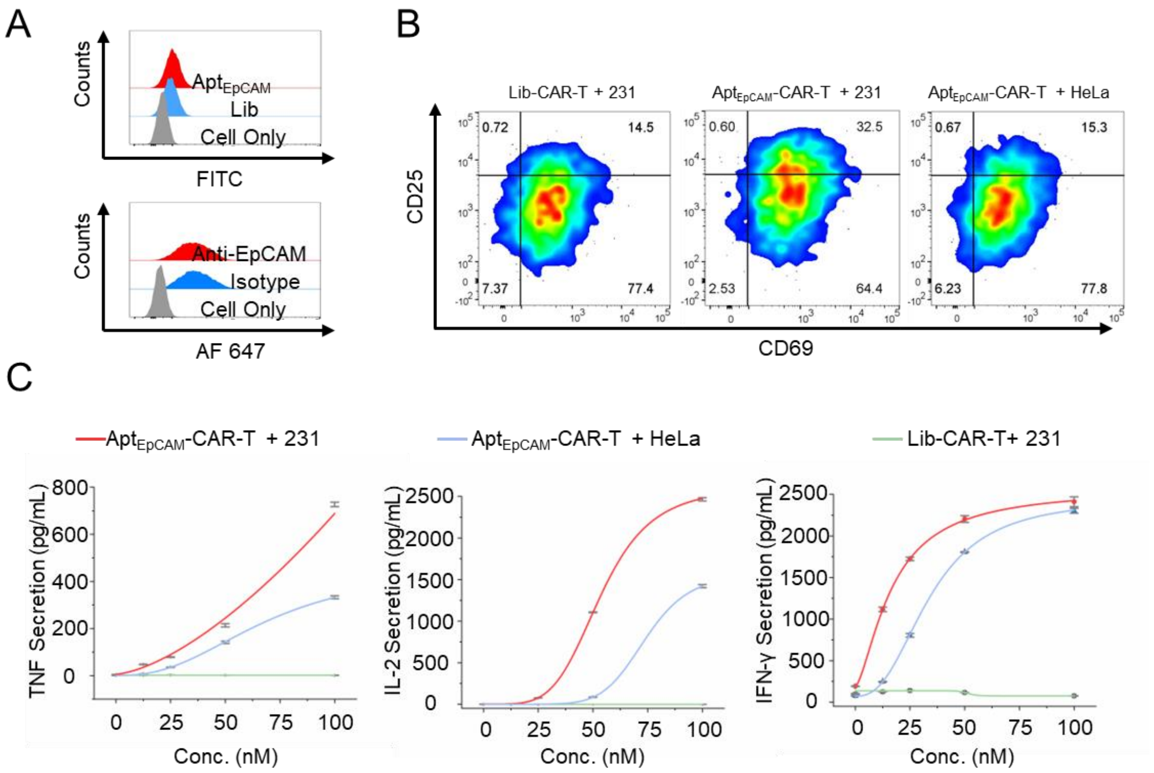

Figure S21. (**A**) Flow cytometry analysis of HeLa cells incubated with 200 nM Lib, Apt_{EpCAM}, Isotype control (1:200) and Anti-EpCAM (1:200) at 4 °C for 30 min. (**B**) CD69 (APC) and CD25 (BV421) expression of Lib-CAR-T, or Apt_{EpCAM}-functionalized CAR-T (Apt_{EpCAM}-CAR-T), after coculture with 231 cells at 37 °C for 24. The concentration of functionalized Lib/Apt_{EpCAM} was 25 nM. (**C**) Cytokine secretion of Lib-CAR-T after coculture with 231 cells at 37 °C for 24 h, or Apt_{EpCAM}-CAR-T after coculture with 231, or HeLa, at 37 °C for 24 h, corresponding with different Lib/Apt_{EpCAM} concentrations. Molar ratio between CAR-T and 231/HeLa cells was fixed at 10: 1. Flow cytometry data are representative data from three independent experiments. All statistical data are presented as the mean value ± S.D., n = 3.

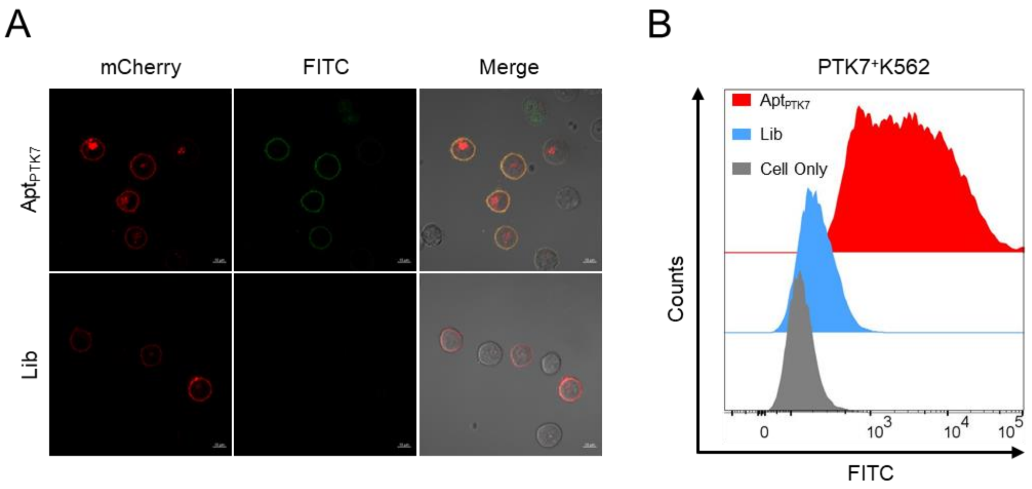

Figure S22. (**A**) CLSM images of PTK7 overexpressed K562 cell lines ($PTK7^+$ K562) binding with 200 nM Apt_{PTK7} and Lib at 4 °C for 30 min. Scale bars represent 10 μm. (**B**) Flow cytometry analysis of PTK7 overexpressed K562 cell lines binding with 200 nM Apt_{PTK7} and Lib at 4 °C for 30 min. These data suggested that mCherry-infused PTK7 overexpressing K562 cell lines had been successfully constructed. Flow cytometry data are representative data from three independent experiments.

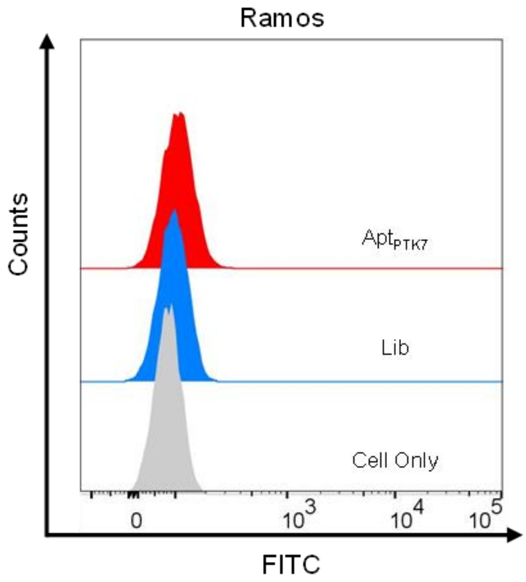

Figure S23. Flow cytometry analysis of Ramos cell lines binding with 200 nM Apt$_{PTK7}$ and Lib at 4 ℃ for 30 min. Data are representative data from three independent experiments.

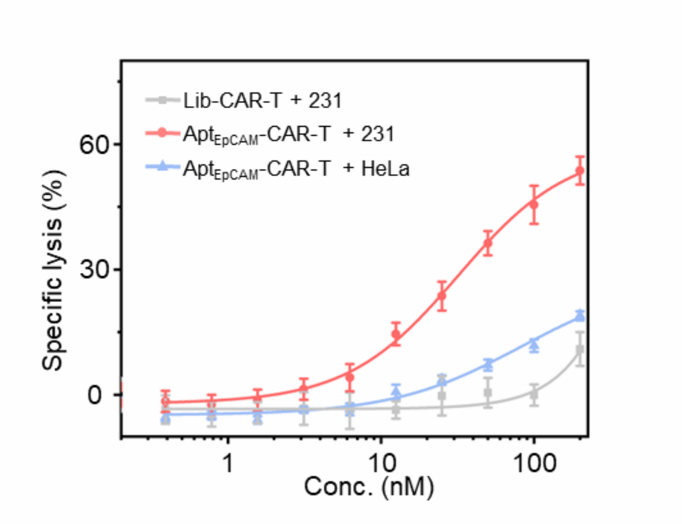

Figure S24. Lysis efficiency of $EpCAM^+$ 231 and $EpCAM^-$ HeLa cells after coculture with Apt_{EpCAM}-CAR-T or Lib-CAR-T at 37 ℃ for 24 h. Molar ratio between CAR-T and 231/HeLa cells was fixed at 10: 1. All statistical data are presented as the mean value ± S.D., n = 3.

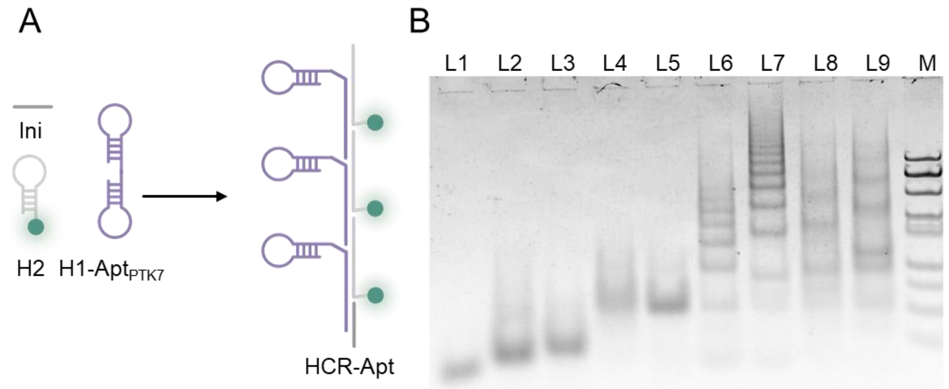

Figure S25. (**A**) Schematic illustration of the design of HCR-Apt. (**B**) PAGE assay (5%) of different DNA samples. From Lane 1 to 9: Ini; H1; H2; H1-Apt$_{PTK7}$; H2-Apt$_{EpCAM}$; Ini + H1 + H2-Lib; Ini + H1-Apt$_{PTK7}$ + H2; Ini + H1 + H2-Apt$_{EpCAM}$; Ini + H1-Apt$_{PTK7}$ + H2-Apt$_{EpCAM}$. Marker represents a 25~500 bp DNA ladder. The concentration of monomer and HCR products was 1 μM and 0.1 μM, respectively. These data suggested that HCR-Apt$_{PTK7}$ and HCR-Apt$_{EpCAM}$ had been successfully constructed.

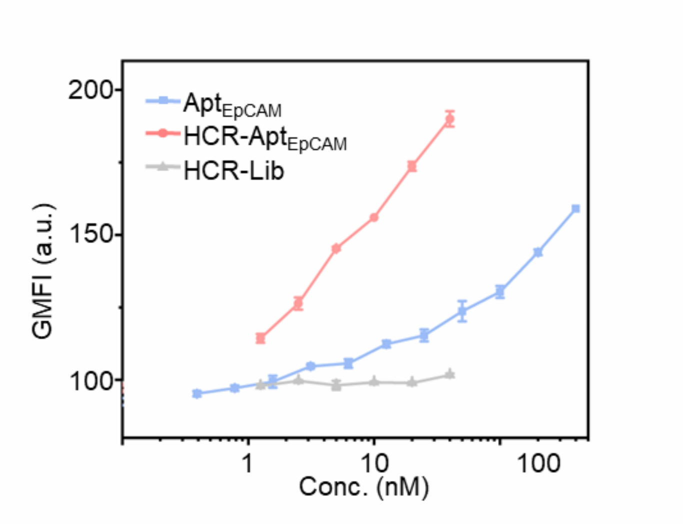

Figure S26. Binding curve of HCR-Apt$_{EpCAM}$, Apt$_{EpCAM}$ and Lib to MDA-MB-231 cells at 4 °C for 30 min. All statistical data are presented as the mean value ± S.D., n = 3.

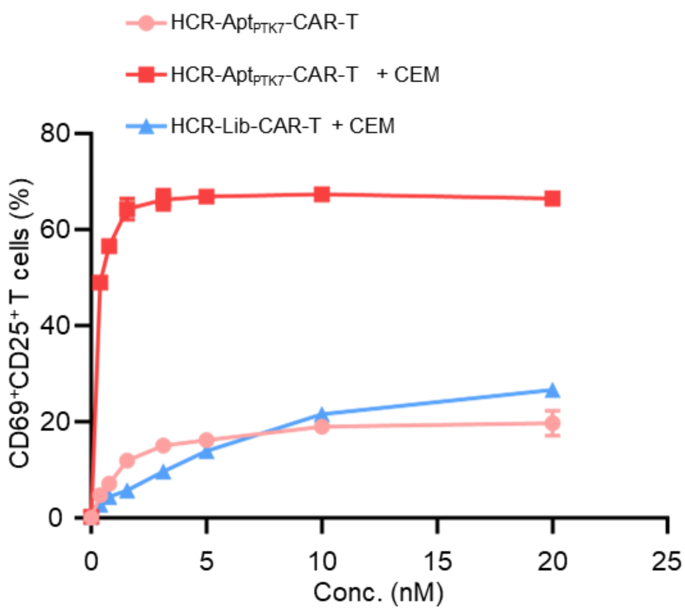

Figure S27. Statistical analysis of CD69 and CD25 expression of HCR-Apt$_{PTK7}$-CAR-T and HCR-Lib-CAR-T after incubation with or without target PTK7^{+} CEM cells at 37 °C for 24 h, as assayed with flow cytometry. Data are presented as the mean value ± S.D., n = 3.

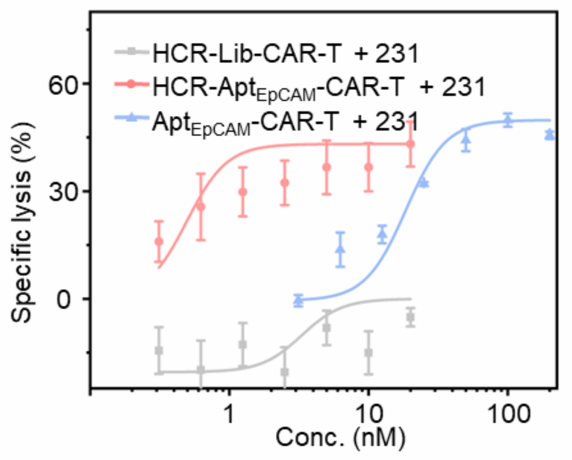

Figure S28. Lysis efficiency of $EpCAM^+$ 231 cells after coculture with Apt-CAR-T functionalized with different concentration of HCR-Lib (HCR-Lib-CAR-T), HCR-Apt_{EpCAM} (HCR-Apt_{EpCAM}-CAR-T) (EC_{50} = 0.49 nM), or Apt_{EpCAM} (Apt_{EpCAM}-CAR-T) (EC_{50} = 18.4 nM) at 37 °C for 24 h. Molar ratio between CAR-T and 231 cells was fixed at 10: 1. All statistical data are presented as the mean value ± S.D., n = 3.

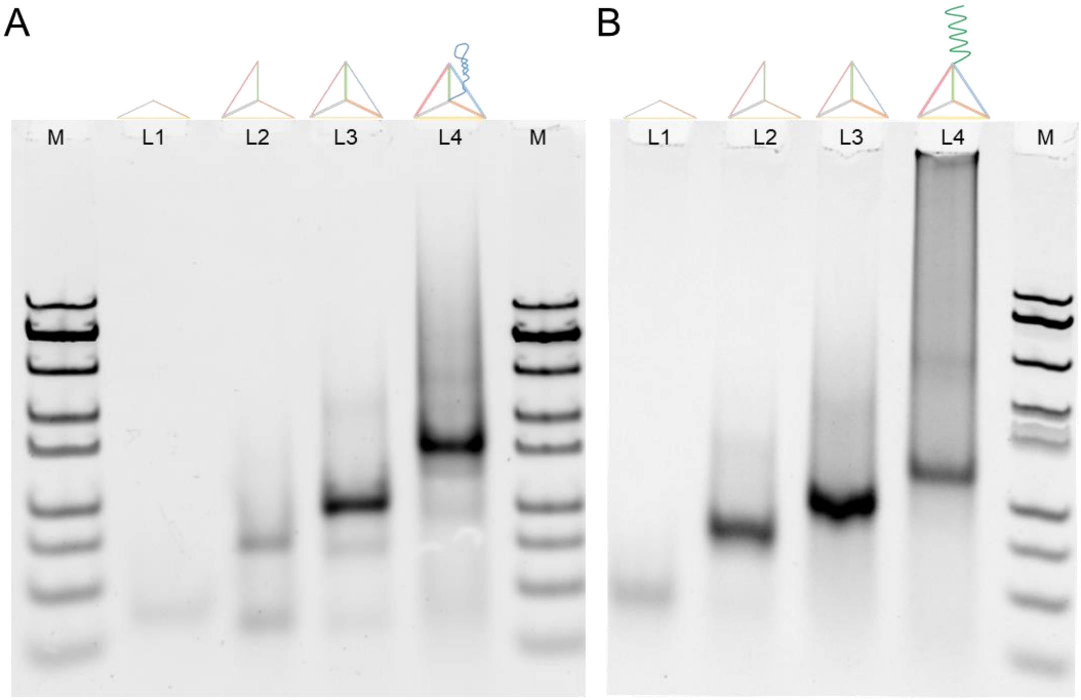

Figure S29. (**A**) Assembly and PAGE (5%) characterization of TDN-Apt$_{PTK7}$. From Lane 1 to 4: A; A + C; A + C + D; A + B-Apt$_{PTK7}$ + C + D. Marker represents a 25~500 bp DNA ladder. The concentration of each sequence is 1 μM. (**B**) Assembly and PAGE (5%) characterization of TDN-Lib. From Lane 1 to 4: A; A + B; A + B + C; A + B + C + D. Marker represents a 25~500 bp DNA ladder. The concentration of each sequence is 1 μM.

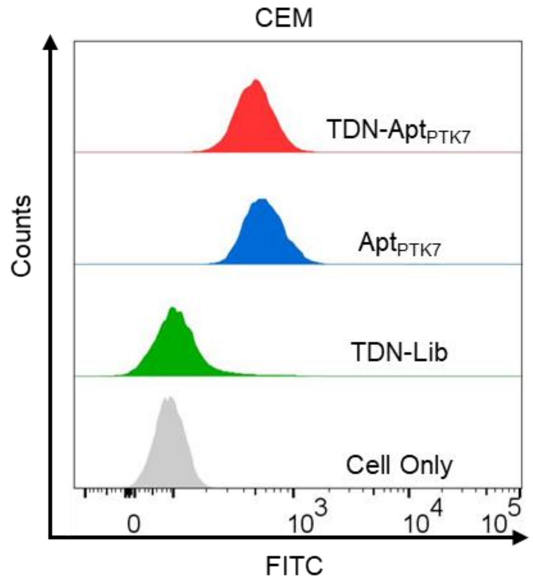

Figure S30. CEM binding with 200 nM TDN-Apt$_{PTK7}$, Apt$_{PTK7}$, or TDN-Lib at 4 °C for 30 min. Data are representative data from three independent experiments.

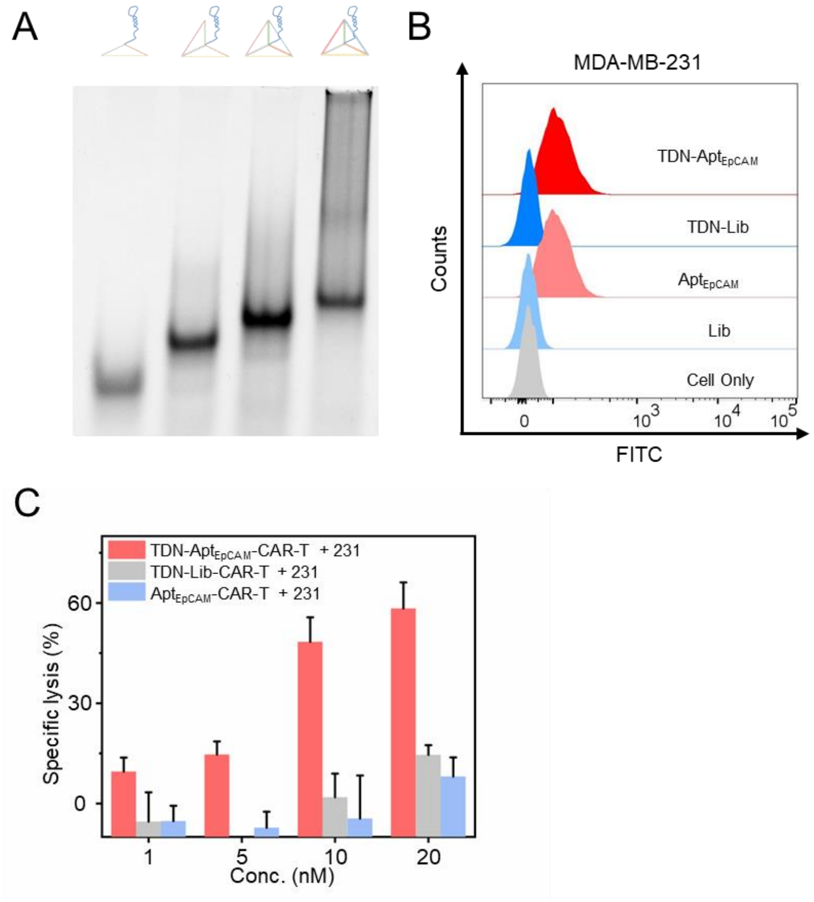

Figure S31. (**A**) Assembly and PAGE (5%) characterization of TDN-Apt$_{EpCAM}$. From Lane 1 to 4: A-Apt$_{EpCAM}$; A-Apt$_{EpCAM}$ + C; A-Apt$_{EpCAM}$ + C + D; A-Apt$_{EpCAM}$ + B + C + D. The concentration of each sequence is 1 µM. (**B**) 231 binding with 200 nM TDN-Apt$_{EpCAM}$, TDN-Lib, Apt$_{EpCAM}$, or Lib at 4 °C for 30 min. (**C**) Lysis efficiency of EpCAM$^+$ 231 cells after coculture with TDN-Apt$_{EpCAM}$-functionalized CAR-T (TDN-Apt$_{EpCAM}$-CAR-T), TDN-Lib-coupled CAR-T (TDN-Lib-CAR-T), or Apt$_{EpCAM}$-CAR-T at 37 °C for 24 h. Molar ratio between CAR-T and 231 cells was fixed at 10: 1. Flow cytometry data are representative data from triplicate samples. All statistical data are presented as the mean value ± S.D., n = 3.

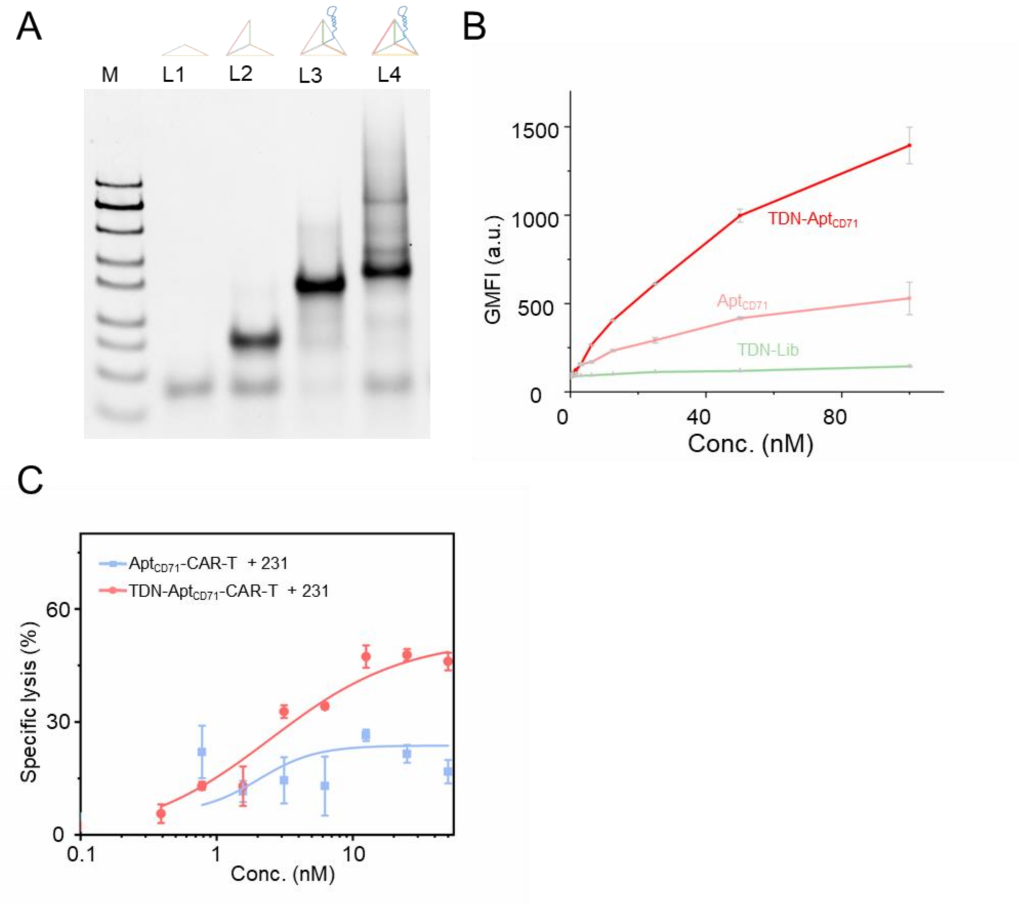

Figure S32. (**A**) Assembly and PAGE (5%) characterization of TDN-Apt$_{CD71}$. From Lane 1 to 4: A; A + B; A + B + C-Apt$_{CD71}$; A + B + C-Apt$_{CD71}$ + D. The concentration of each sequence is 1 μM. (**B**) CEM binding curve with different concentration of TDN-Apt$_{CD71}$, TDN-Lib, or Apt$_{CD71}$ at 4 °C for 30 min. (**C**) Lysis efficiency of CD71^{+} CEM cells after coculture with TDN-Apt$_{CD71}$-functionalized CAR-T (TDN-Apt$_{CD71}$-CAR-T) or Apt$_{CD71}$-functionalized CAR-T (Apt$_{CD71}$-CAR-T) at 37 °C for 24 h. Molar ratio between CAR-T and CEM cells was fixed at 10: 1. All statistical data are presented as the mean value ± S.D., n = 3.

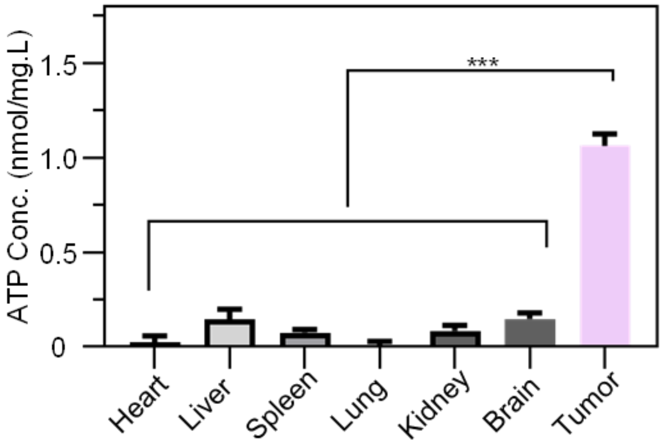

Figure S33. The ATP concentration in main organs of 231 cell-based tumor-bearing mice, as assayed with enhanced ATP Assay Kit and normalized with the weight of corresponding organs. All statistical data are presented as the mean value ± S.D., based on data obtained from three tumor-bearing mice. *$P \leq 0.05$, **$P \leq 0.01$, ***$P \leq 0.001$, and ****$P \leq 0.0001$ by two-tailed Student's t-test.

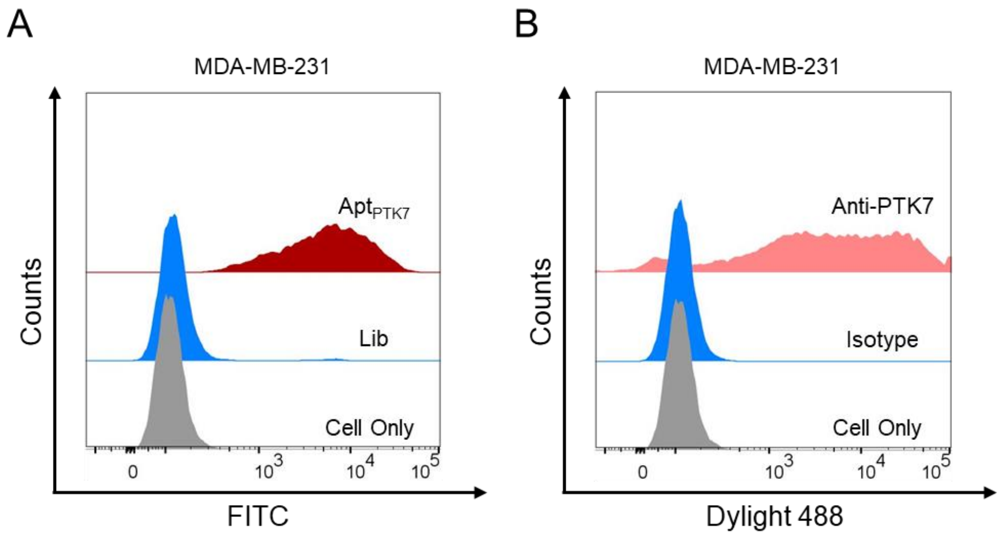

Figure S34. (**A**) Flow cytometry analysis of MDA-MB-231 cells incubated with 200 FITC-labeled nM Lib or Apt$_{PTK7}$ in binding buffer at 4 °C for 30 min. (**B**) Flow cytometry analysis of MDA-MB-231 cells incubated with Dylight 488-conjugated anti-PTK7 antibody or isotype control (1:200) in binding buffer at 4 °C for 30 min. Flow cytometry data are representative data from three independent experiments.

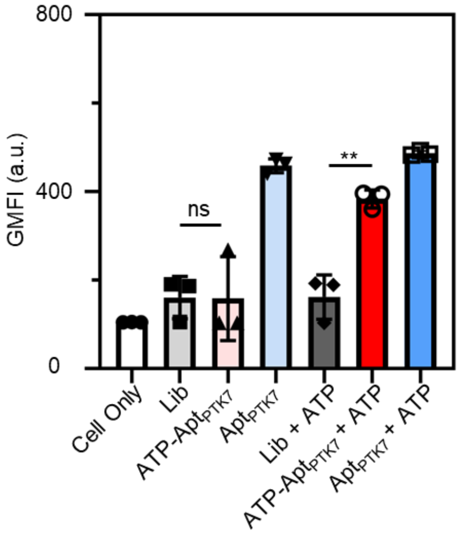

Figure S35. Flow cytometry of 231 cells after incubation with 200 nM Lib, ATP-Apt$_{PTK7}$, and Apt$_{PTK7}$ at 4 ^{o}C for 30 min in the presence (+) and absence (-) of 1 mM ATP. This result represents the statistical analysis of the flow cytometry data shown in Figure 4B. Data are presented as the mean value ± S.D., n = 3. *P ≤ 0.05, **P ≤ 0.01, ***P ≤ 0.001, and ****P ≤ 0.0001 by two-tailed Student's t-test.

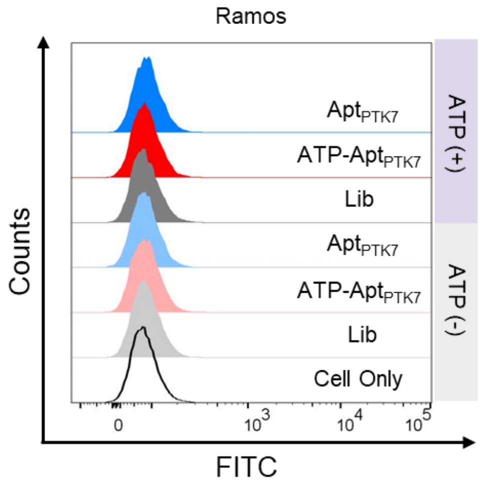

Figure S36. Flow cytometry of Ramos cells after incubation with 200 nM FITC-labeled Lib, ATP-Apt$_{PTK7}$, and Apt$_{PTK7}$ at 4 °C for 30 min in the presence (+) and absence (-) of 1 mM ATP. Flow cytometry data are representative data from three independent experiments.

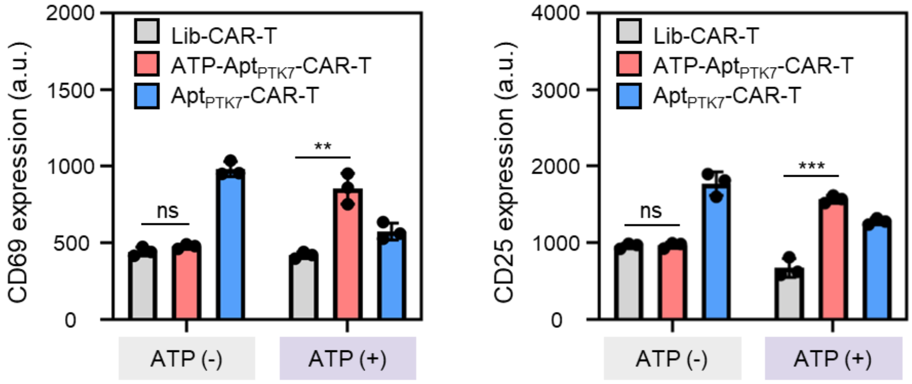

Figure S37. Statistical analysis of CD69 and CD25 expression of Lib-CAR-T, ATP-Apt$_{PTK7}$-functionalized CAR-T (ATP-Apt$_{PTK7}$-CAR-T), or Apt$_{PTK7}$-CAR-T after coculture with target PTK7$^+$ 231 cells at 37 °C for 24 h in the presence (+) and absence (-) of 62.5 μM ATP. Data are presented as the mean value ± S.D., n = 3. *P ≤ 0.05, **P ≤ 0.01, ***P ≤ 0.001 and ****P ≤ 0.0001 by two-tailed Student's t-test.

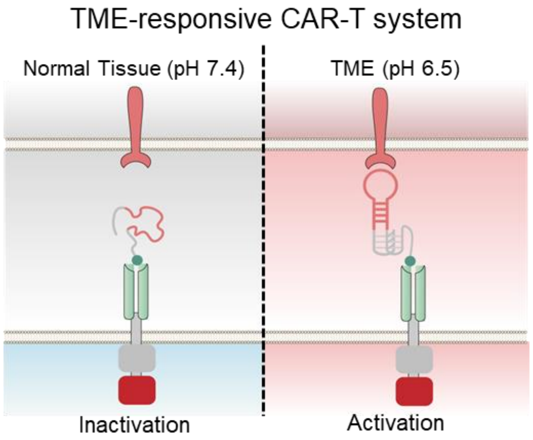

Figure S38. Schematic illustration of the tumor microenvironment (low pH)-responsive antigen targeting strategy of CAR-T.

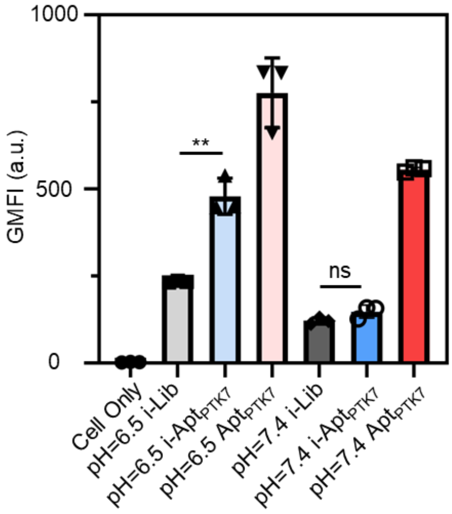

Figure S39. Flow cytometry of 231 cells after incubation with 200 nM Cy5-labeled i-Lib, i-Apt$_{PTK7}$, and Apt$_{PTK7}$ at 4 ^{o}C at pH 6.5 or pH 7.4 for 30 min. This result represents the statistical analysis of the flow cytometry data shown in Figure 4E. Data are presented as the mean value ± S.D., n = 3. *P ≤ 0.05, **P ≤ 0.01, ***P ≤ 0.001, and ****P ≤ 0.0001 by two-tailed Student's t-test.

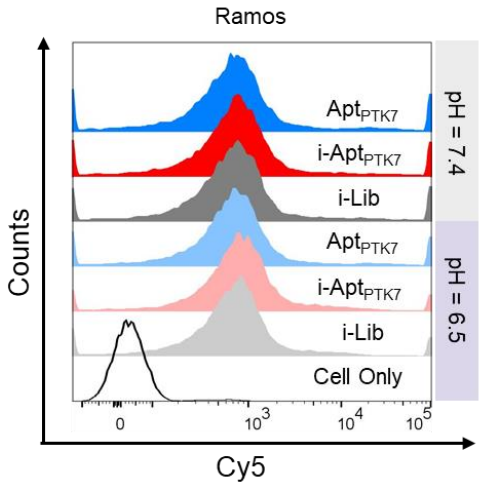

Figure S40. Flow cytometry of Ramos cells after incubation with 200 nM FITC-labeled i-Lib, i-Apt$_{PTK7}$, and Apt$_{PTK7}$ at pH 6.5 or pH 7.4 at 4 ^{o}C for 30 min. Flow cytometry data are representative data from three independent experiments.

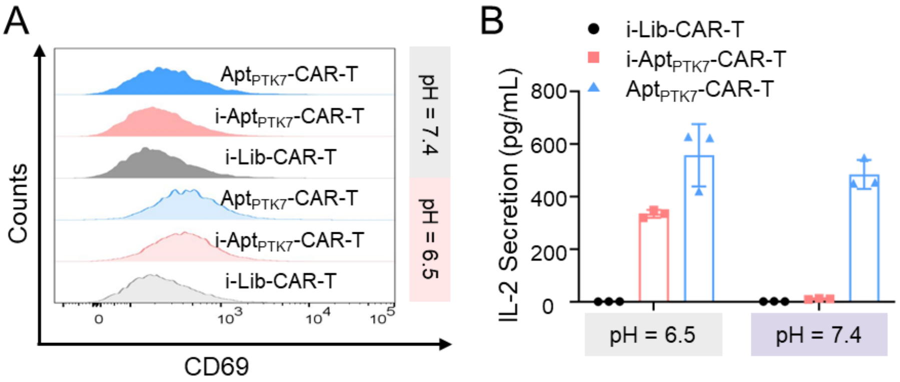

Figure S41. (**A**) CD69 (APC-conjugated antibody) expression of i-Lib-CAR-T, i-Apt_{PTK7}-functionalized CAR-T (i-Apt_{PTK7}-CAR-T), or Apt_{PTK7}-CAR-T after coculture with target $PTK7^{+}$ 231 cells at pH 6.5 or pH 7.4 for 24 h, as assayed with flow cytometry. Flow cytometry data are representative data from three independent experiments. (**B**) IL-2 secretion of $PTK7^{+}$ 231 cells after coculture with i-Lib-CAR-T, i-Apt_{PTK7}-CAR-T, or Apt_{PTK7}-CAR-T at pH 6.5 and 7.4 for 24 h. All statistical data are presented as the mean value ± S.D., n = 3.

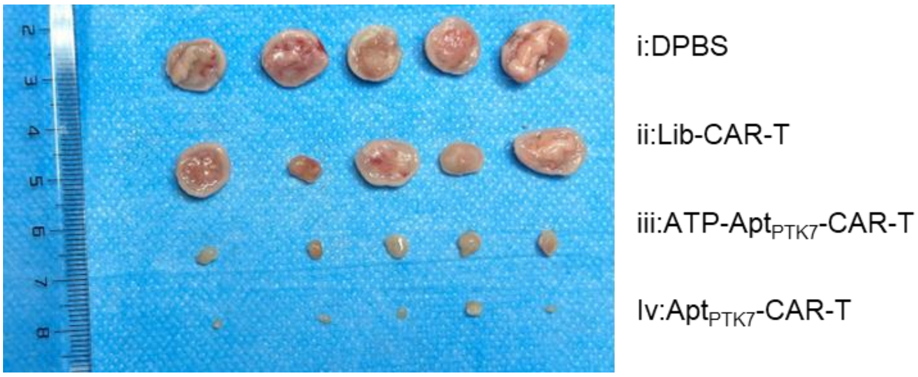

Figure S42. Image of PTK7^{+} 231 tumor harvested from mice 42 days post-specific treatments.

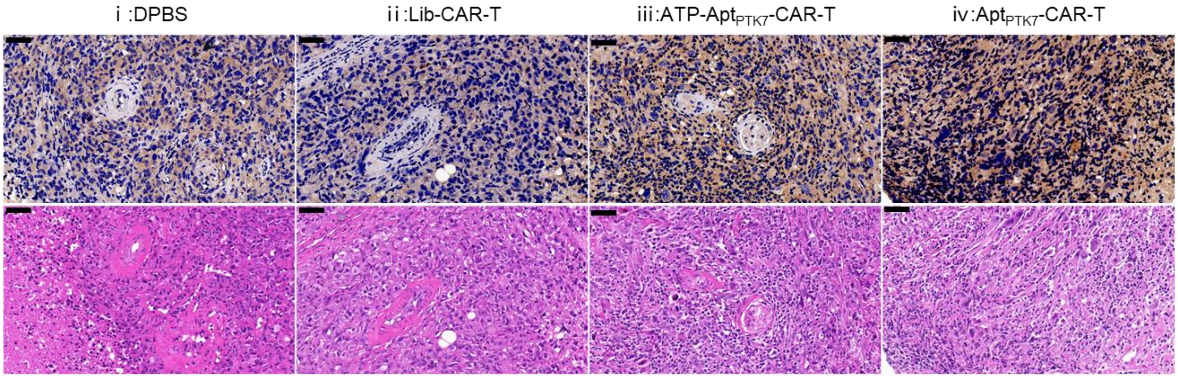

Figure S43. Immunohistochemical staining assay (upper panel) and H&E staining assay (lower panel) assay of tumor tissues harvested from mice 42 days post-specific treatments. Scale bar: 50 μm.

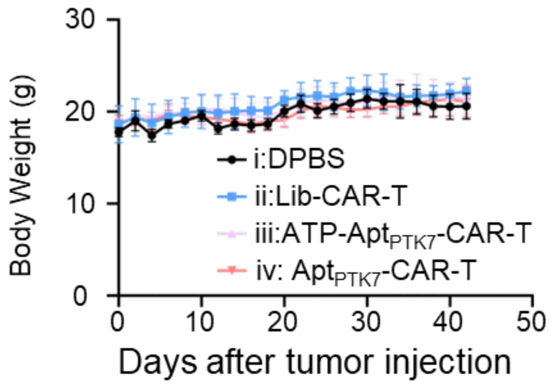

Figure S44. Average body weight (n=5) curves of tumor-bearing mice processed with different treatments. Statistical data are presented as the mean value ± S.D., n = 5.

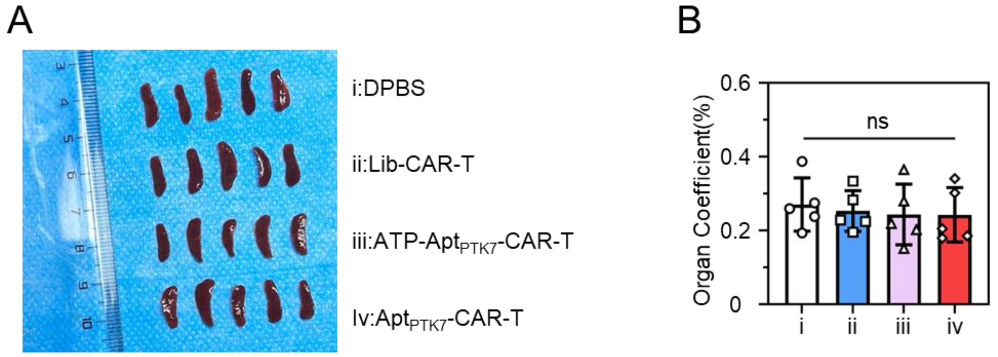

Figure S45. Images and organ coefficient analysis of spleen (n = 5) harvested from mice 42 days post-specific treatments. Statistical data are presented as the mean value ± S.D., n = 5.

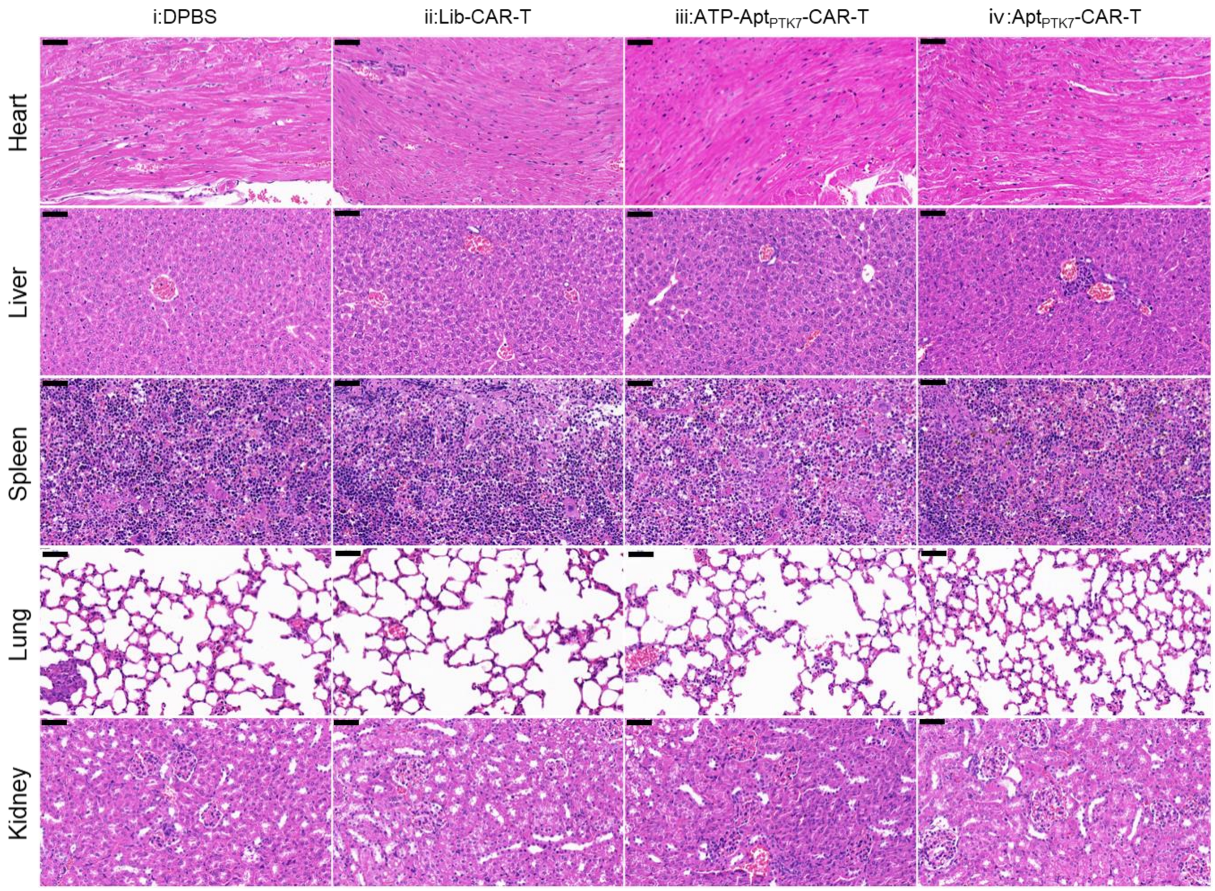

Figure S46. H&E assay of main organs harvested from mice 42 days post-specific treatments. Scale bar: 50 μm.

Reference

[1] Ma, J. S. Y.; Kim, J. Y.; et al. Versatile strategy for controlling the specificity and activity of engineered T cells. *Proc. Natl. Acad. Sci. U.S.A.* **2016**, *113*, E450-E458.
